# Perennials as Future Grain Crops: Opportunities and Challenges

**DOI:** 10.3389/fpls.2022.898769

**Published:** 2022-07-29

**Authors:** Elizabeth A. Chapman, Hanne Cecilie Thomsen, Sophia Tulloch, Pedro M. P. Correia, Guangbin Luo, Javad Najafi, Lee R. DeHaan, Timothy E. Crews, Lennart Olsson, Per-Olof Lundquist, Anna Westerbergh, Pai Rosager Pedas, Søren Knudsen, Michael Palmgren

**Affiliations:** ^1^Department of Raw Materials, Carlsberg Research Laboratory, Copenhagen, Denmark; ^2^Department of Plant and Environmental Sciences, University of Copenhagen, Frederiksberg, Denmark; ^3^The Land Institute, Salina, KS, United States; ^4^Lund University Centre for Sustainability Studies, Lund, Sweden; ^5^Department of Plant Biology, Uppsala BioCenter, Linnean Centre for Plant Biology in Uppsala, Swedish University of Agricultural Sciences, Uppsala, Sweden

**Keywords:** breeding, domestication, genome editing, grain crops, perennialism, species-wide hybridization, perennial grains, perennial agriculture

## Abstract

Perennial grain crops could make a valuable addition to sustainable agriculture, potentially even as an alternative to their annual counterparts. The ability of perennials to grow year after year significantly reduces the number of agricultural inputs required, in terms of both planting and weed control, while reduced tillage improves soil health and on-farm biodiversity. Presently, perennial grain crops are not grown at large scale, mainly due to their early stages of domestication and current low yields. Narrowing the yield gap between perennial and annual grain crops will depend on characterizing differences in their life cycles, resource allocation, and reproductive strategies and understanding the trade-offs between annualism, perennialism, and yield. The genetic and biochemical pathways controlling plant growth, physiology, and senescence should be analyzed in perennial crop plants. This information could then be used to facilitate tailored genetic improvement of selected perennial grain crops to improve agronomic traits and enhance yield, while maintaining the benefits associated with perennialism.

## Introduction

Climate change has led to an increased focus on sustainable agricultural practices to produce food and feed using less energy and with a lower carbon footprint (Batello et al., 2013; IPCC, 2019). For millennia, the repeated selection and breeding of plants has led to the development of multiple, high-yielding annual grain crops finely tuned for growth under specific environmental regimes. In the twentieth century, cropping systems were developed that took advantage of readily available resources and agrichemical development, with the focus primarily on grain yield. However, considering the current range of complex challenges that agriculture faces, including climate change, pandemics, and war, the focus must now be on ensuring food security in a more environmentally friendly and socially robust way (Beddington et al., 2012). Continued climate change is rendering our existing cultivars increasingly vulnerable to stress, and ultimately unfit for many regions of the world, serving as another impetus for reinventing agriculture (Altieri et al., 2015; Asseng et al., 2015). Such a shift necessitates reduced fertilizer and pesticide application, adaptation and adoption of plant genetic variation, and reduced tillage, which together would lower a crop's carbon footprint. These measures can be applied to all major crops grown today, but could also involve alternative cropping systems, as suggested by DeHaan et al. (2005) and Glover et al. (2010).

Grain crops can be divided into two broad types, namely, annuals and perennials, based on when their life cycles terminate (Friedman, 2020). Annual plants grow for one season, produce seed, initiate senescence, and die. Annualism is linked to seed dormancy traits, i.e., under conditions of environmental stress, the seeds become part of the soil seed bank, with dormancy broken upon return of favorable conditions. Thus, annuals have a short juvenile phase coupled with rapid seed production that favors species survival (Lundgren and Des Marais, 2020). At present, all the main cereal grain crops are annuals, requiring annual replanting and cultivation. Worldwide, maize (*Zea mays*), rice (*Oryza sativa*), and wheat (*Triticum aestivum*) account for 28% of global primary crop production (FAO, 2021). However, while annual crops deliver high yields, extensive tilling, field preparation, crop management, and routine agrichemical application are required for optimal outcomes.

**Figure 1:**
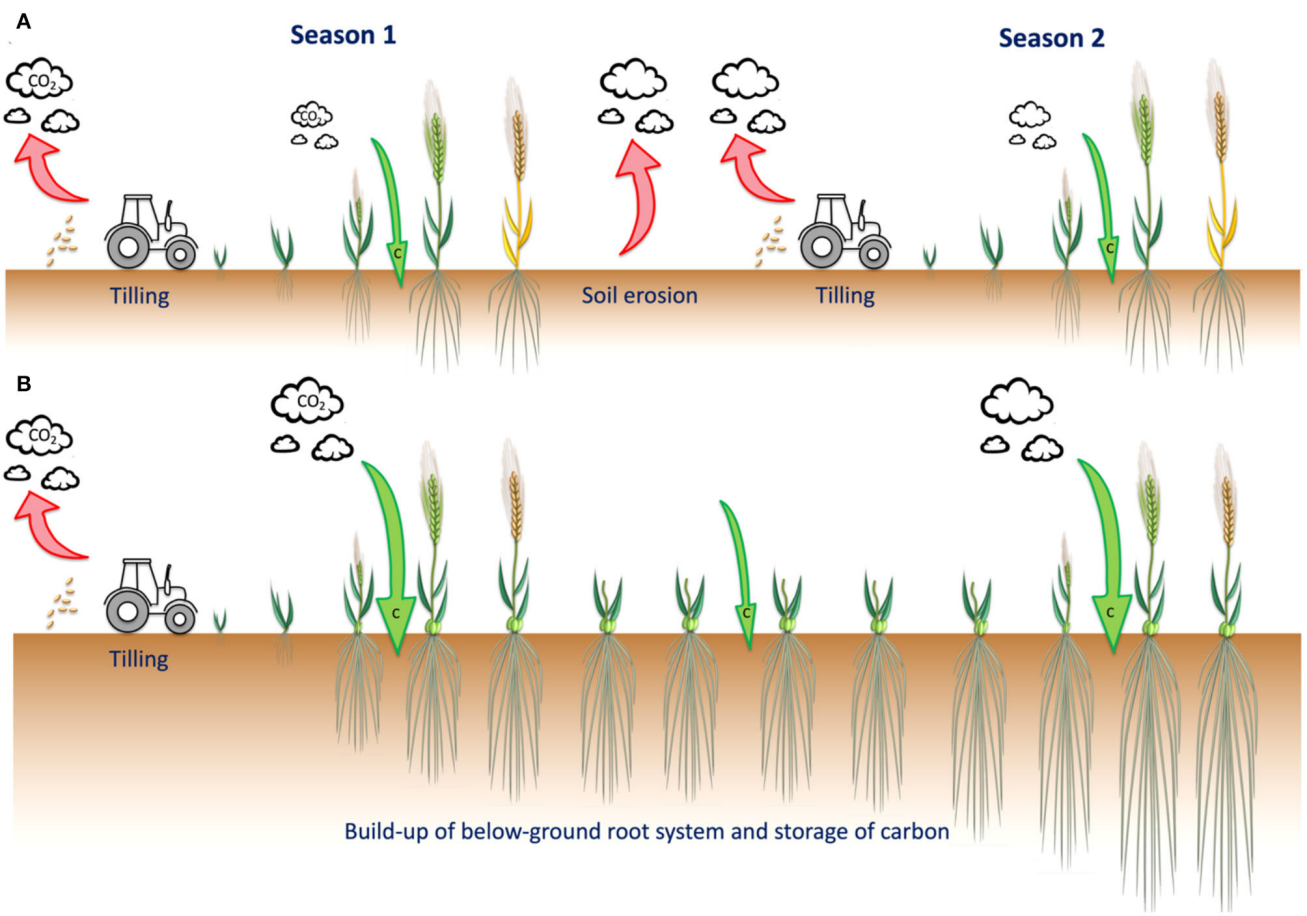
A perennial grain crop would offer a sustainable alternative to present day annual crops. **(A)** Annual crops live only for a single season with annual cultivation dependent on machines for tilling and sowing. In addition to promoting soil erosion, tillage breaks open soil aggregates, exposing the previously protected organic matter to microbes resulting in elevated respiration and losses of CO_2_ to the atmosphere. **(B)** Perennial crops need tilling and sowing only in the first year and thereafter are viable for several seasons. Some perennial grain crops require vernalization, and are typically autumn sown with grain harvested in the following years. Perennial grain crops develop an extensive root system that stores carbon underground, and also grows during cool periods of the year. Depending on the cropping history and management, both annual and perennial crops can contribute to soil carbon sequestration, with the contribution of perennial grain crops significantly greater due to the reduction in tilling and greater allocation of photosynthates to root systems over time. Such differences are illustrated using barley *H. vulgare* (annual) and *H. bulbosum* (perennial) as examples.

On the contrary, in natural ecosystems, perennial plant species dominate net primary productivity; defined as the difference between energy fixed by autotrophs and their respiration (Díaz, 2001). Thus, in almost every major terrestrial biome, including forests, grasslands, savannahs, deserts, and tundra, annuals tend to be outcompeted by perennials (Tilman, 1988). In agriculture, many environmental benefits are associated with perennialism. Once planted, perennial crops can be grown for several seasons and harvested annually, reducing the need for tillage, while their deep rooting habits help to actively increase soil carbon over time (Figure 1; Paustian et al., 2016; Ledo et al., 2020; Peixoto et al., 2022). Perennialism is also associated with greater nutrient uptake, environmental resilience, weed suppression, reduced soil erosion and nutrient leaching, and increased biomass of soil microbial communities, highlighting the potential contribution of perennial crops to no-till agriculture (Lundgren and Des Marais, 2020; Audu et al., 2022; Soto-Gómez and Pérez-Rodríguez, 2022). From a social and economic perspective, perennial grain crops have the potential to improve rural economies through reducing the need for costly external inputs and labor intensity (Crews et al., 2018).

## Comparative Physiology of Annuals and Perennials

The perennial habit is associated with a wide range of physiological traits, likely necessitated by the greater range of environmental and seasonal cues encountered by these plants compared to their annual counterparts (Lundgren and Des Marais, 2020).

**Figure 2:**
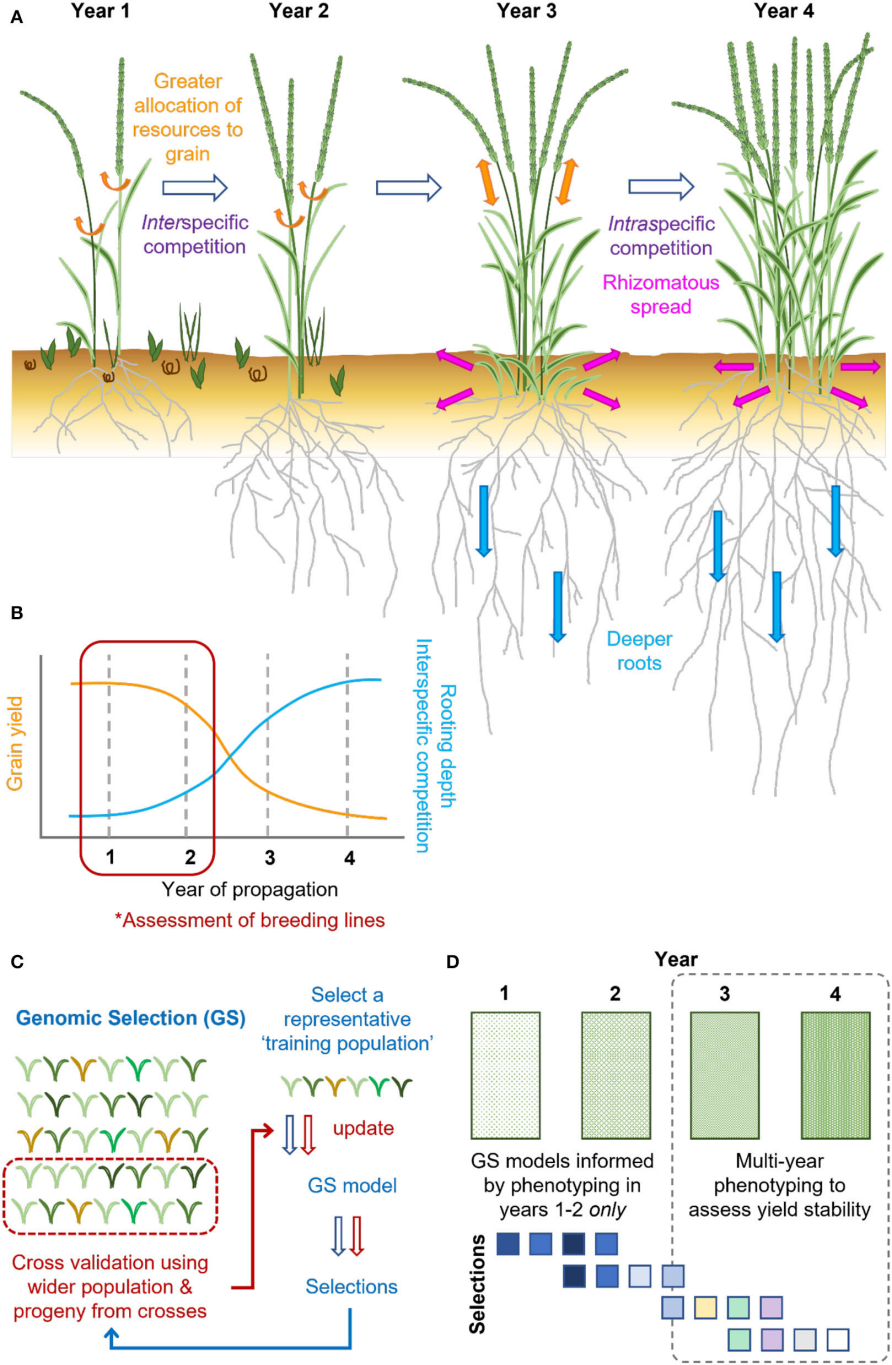
Grain yield of intermediate wheatgrass (*T. intermedium*) varies over time, which existing genomic selection practices need to be designed to overcome. **(A)** Grain yield of intermediate wheatgrass is typically greatest in the first 2 years of propagation, with stem reserves and photosynthates allocated toward the grain (orange arrows). As plantings establish, intermediate wheatgrass outcompetes weeds (interspecific competition), with plants increasingly competing among themselves (intraspecific competition). Over time, the greater resources are allocated toward clonal growth and rhizomatous spread (pink arrows), in place of grain production (orange arrows), alongside deeper rooting (blue arrows), facilitating overwintering and survival. **(B)** The declining grain yields of intermediate wheatgrass over time are associated with differential resource allocation. However, phenotyping conducted in 1–2 years fails to capture this. **(C)** Genomic selection (GS) is routinely applied in intermediate wheatgrass breeding. GS relies on the creation of a training set representative of the genotypic and phenotypic variation found within the wider breeding program. Using an iterative approach, phenotypic and genotypic data are integrated to identify trait genetic associations to predict future plant performance (arrows indicate stages, blue = 1, red = 2). **(D)** In absence of multi-year phenotyping (dashed line; * in B) existing GS pipelines cannot select for yield stability. Additional phenotyping of the same plant stands in 3–4 years (represented by increasingly green rectangles) would help address this, for which there may be little consensus between the earlier, and these newer, selections (different colored squares).

Perennial species live for many years, with most plants cycling back and forth between growth and reproduction over multiple seasons (Figure 1). During the first growing season, seeds are sown, germinate, and plants undergo a juvenile phase, with growth in the subsequent seasons initiated from both the crown and their extensive available root systems (Figure 2A; Thomas et al., 2000; Friedman and Rubin, 2015). One distinguishing feature between annual and perennial grass species is the ability of perennials to produce vegetatively propagating organs, such as the rhizomes of perennial wild rice (*Oryza longistaminata*; Guo et al., 2021), wild sorghum (*Sorghum halepense*; Paterson et al., 2020), and intermediate wheatgrass (*Thinopyrum intermedium*; Figure 2A; DeHaan et al., 2020), or the bulbous structure of the perennial grass species *Hordeum bulbosum* (Westerbergh et al., 2018).

Underlying the distinct life cycles of annual and perennial plants are differences in resource allocation. Annuals predominantly direct their resources toward reproduction, while perennials support both maintenance of vegetative organs and seed production (Figure 1). The high grain yields and improved agronomic performance of domesticated annual cereals (Harlan et al., 1973; Kantar et al., 2016) results from the significant re-allocation of carbon during monocarpic senescence, the final stage of the plant's life cycle. Therefore, the fast growth and high reproductive effort of annuals contribute to their high yielding ability. In contrast, the delayed reproduction and long-term survival strategy of perennials may reduce resources allocated to reproduction *via* seed or grain (Friedman, 2020). Despite this, the pattern of resource allocation can vary, with some perennial species displaying fast growth and high reproductive output (Verboom et al., 2004; González-Paleo and Ravetta, 2015), as exemplified by the tropical perennial herbaceous crops enset (*Ensete ventricosum*) and banana (*Musa sp*.), which can produce 50 t ha^−1^ of starchy fruit per year (Kreitzman et al., 2020). Consequently, a better understanding of the mechanisms underlying such specific adaptations could aid adaptation to specific environments, for which the manipulation of reproductive phases to increase environmental synchronicity could improve agronomic performance and grain yield.

During monocarpic senescence in wheat, 80% of leaf nitrogen and phosphorus is remobilized and reassimilated into the developing grain, with leaves maintained until process completion (Buchanan-Wollaston, 2007). Senescence is subject to tight genetic and environmental control, ensuring synchronicity of a plant's life cycle with changing seasons and environmental stress. In wheat, delayed senescence is associated with extending grain fill duration, positively influencing grain yield (Spano et al., 2003; Pinto et al., 2016; de Souza Luche et al., 2017; Chapman et al., 2021). Furthermore, among the annual grain crops such as maize, rice, barley (*Hordeum vulgare*), and sorghum (*Sorghum bicolor*), delays in senescence or “staygreen” traits are associated with stress tolerance (Gregersen et al., 2013; Thomas and Ougham, 2014). In perennial species, senescence coincides with decreasing daylength and temperatures, with plants effectively shutting down for winter, reallocating resources to facilitate their overwintering and survival (Figure 2A; Lundgren and Des Marais, 2020).

## The Trade-Off Between Perennialism and Yield

Any trait considered advantageous may have a price and represent a trade-off with respect to another trait. This raises the question, “What is the cost of perennialism in terms of yield?” In an evolutionary sense, there need not be constraint between the two traits even though natural selection never favored evolution of both traits in a species (Garland, 2014). However, even when reproductive allocation *vs*. longevity has a trade-off in a wild species, life history strategies that develop under natural selection may not necessarily be predictive of trade-offs under artificial selection. For example, breeding of annual grain crops has been effective in selecting for reduced competitive ability (Reynolds et al., 1994). In the same way, breeding is expected to increase grain yield of perennials through the reduction in allocation towards their competitive ability (Figure 2B; DeHaan et al., 2005). Since some perennial crops can have capacity for above-ground biomass production similar to, or greater than, annual crops (Dohleman and Long, 2009), there might not be a trade-off between longevity and yield.

In cereals, delayed senescence can be associated with physiological differences, akin to those observed between annual grain crops and their perennial counterparts, with both representing trade-offs regarding resource allocation. For example, in annual sorghum (*S. bicolor*), staygreen traits are associated with reduced tillering, deeper roots, altered stomatal conductance, and improved nitrogen dynamics (Borrell et al., 2003, 2014). In annual cereal grain crops, staygreen traits can be associated with a reduction in nitrogen remobilization efficiency. This represents a trade-off between maintenance of photosynthetic tissue and re-allocation of resources to the grain, potentially negatively impacting grain yield. For example, Borrill et al. (2015) reported that the additional photosynthates of *NAM* RNAi wheat (*T. aestivum*) plants were stored as stem fructan and not remobilized. Similarly, a study in maize (*Z. mays*) found that while post-anthesis nitrogen uptake of staygreen lines was greater compared to non-staygreen types, nitrogen remobilization to the grain was lower (Chibane et al., 2021). However, in perennial cereals, such stored nitrogen may potentially be available for re-allocation in subsequent years (Crews et al., 2016).

In perennial grain crops, a fine balance must be met between allocation of resources to harvestable grain and seasonal survival (Lundgren and Des Marais, 2020). This includes maintenance of rhizomes, bulbous structures, stolons, or dormant tiller buds that emerge from old tillers, from which plants regrow in the coming year following senescence and overwintering (Figure 2A; Palmer et al., 2015; Lindberg et al., 2020; Lundgren and Des Marais, 2020). Leaf anatomy of perennials and annuals can differ, with substantial dynamism reported during the plant's lifespan, particularly in response to stress (Jaikumar et al., 2016; Lundgren and Des Marais, 2020). For example, under cold stress, the photosynthetic rate and Rubisco concentration of 5-year-old intermediate wheatgrass (*T. intermedium*) plants were significantly greater when compared to 2-year-old plants (Jaikumar et al., 2016). In contrast, overall net daily rate of photosynthesis of perennial *H. bulbosum* was less than two-thirds that of annual *H. vulgare* (Burnett et al., 2016). The balance between grain production, survival, and vegetative growth of intermediate wheatgrass also shifts as plantings age (DeHaan et al., 2013; Tautges et al., 2018), as illustrated by the 45–80% lower grain yields of 3 *vs*. 1-year-old plant stands (Hunter et al., 2020; Figures 2A,B).

In intermediate wheatgrass, the significant reduction in grain yield over time is likely attributable to increased intraspecific competition as plants spread over time and with greater carbon accumulation within the root zone (Figure 2B; Law et al., 2021). Consequently, Lundgren and Des Marais (2020) suggest these larger root systems compete for available photosynthates, as evident when comparing *Bromus* and *Lupinus* annual–perennial species pairs. To balance these competing demands, mechanical means could be adopted to reduce tillering of older stands, thereby reducing intraspecific competition (Law et al., 2021) and potentially redirecting root reserves.

A short lifespan of a plant species, whereby the age of reproductive maturity is reached at an earlier stage, may allow the species to adapt faster to changing conditions. For this reason, ignoring other factors, it has been proposed that annual species could adapt more rapidly than perennials to the more extreme and the unpredictable environments associated with climate change (Jump and Peñuelas, 2005; Berger et al., 2016; Norton et al., 2016; Lundgren and Des Marais, 2020). Thus, there may be potential trade-off between perennialism and adaptive capacity. However, although generation time was a constraint that has in the past limited progress in the breeding of perennials, modern technology is providing many short-cuts. Thus, marker-assisted selection and more recently, genomic selection has enabled rapid cycling of generations in perennials (McClure et al., 2014; Crain et al., 2021a,b). Lately, with the advent of genome editing, generation time of the edited plant may even become irrelevant. Available technologies for accelerated breeding and domestication will be discussed further below in this review.

## The Differing Mating Habits of Perennials and Annuals

Within the Poaceae, self-incompatibility (SI) is widespread and generally more prevalent in perennial crops compared to annuals (Baumann et al., 2000). Many of the perennial relatives of common grain crop species are self-incompatible and outcrossing (Table 1). Self-incompatibility reduces the ability to readily create homozygous and inbred lines, as is standard practice during breeding of barley, rice, and wheat, and can reduce seed set owing to pollen limitation (Aizen and Harder, 2007). Consequently, reducing or converting promising perennial grain crop species from being self-incompatible to self-compatible, as achieved during domestication of sunflower (*Helianthus annuus*) (Liu and Burke, 2006), is a key breeding target.

**Table 1:** Comparison of mating type and ploidy level of annual grain crops and their perennial relatives for which interspecific crosses have been performed.

**Annual**	**Mating type**	**Ploidy**	**Perennial**	**Mating type**	**Ploidy**	**Traits mapped (QTLs)**	**Study**
Barley (*H. vulgare*)	Inbreeding	2*n* = 2*x* = 14	*H. bulbosum*	Outcrossing	2n = 2*x* = 14; 2n = *4x* = 28	–	
Wheat (*T. aestivum*)	Inbreeding	2*n* = 6*x* = 42	*Thinopyrum* species	Outcrossing	2*n* = 2*x* = 14 to 2n = 10*x* = 70	–	
Rice (*O. sativa*)	Inbreeding	2*n* = 2*x* = 24	*Oryza rufipogon, Oryza rhizomatis, O. longistaminata, Oryza officinalis, Oryza australiensis*	Outcrossing	2*n* = 2*x* = 24	Cold tolerance (*qCT2.1*), rhizatomous growth (*Rhz2, Rhz3*) Rhizome development (*qRED1.2, qRED3.1, qRED3.3, qRED4.1, qRED4.2*)	Hu et al., 2003, 2011; Sacks et al., 2008; Fan et al., 2020; Yuan et al., 2020
Rye (*Secale cereale*)	Outcrossing	2*n* = 2*x* = 14	*Secale strictum* (*S. monatum*)	Outcrossing	2*n* = 2*x* = 14	Perenniality (*QTL-P2, QTL-P3, QTL-P4, QTL-P5, QTL-P7*)	Gruner and Miedaner, 2021
Sorghum (*S. bicolor*)	Predominantly inbreeding	2*n* = 2*x* = 20	*S. halepense, Sorghum propinquum*	Predominantly inbreeding	2*n* = 4*x* = 40	Rhizomatousness, overwintering, height and flowering time, dwarfing (*Dw1, Dw3*), dry stalk (*D*), maturity, biomass yield	Washburn et al., 2013; Cox et al., 2018; Habyarimana et al., 2020; Kong et al., 2021
Maize (*Z. mays*)	Outcrossing	2*n* = 2*x* = 20	*Zea diploperennis, Zea perennis*	Outcrossing	2*n* = 2*x* = 20; 2*n* = 4*x* = 40	Rhizome formation, tillering, staygreen, perennial regrowth (*reg1, reg2, reg3*)	Westerbergh and Doebley, 2004; Coatney, 2015; Ma et al., 2019; Swentowsky et al., 2021

*In many cases, interspecific crosses have facilitated QTL mapping of perennial traits. Quantitative trait locus mapping could not be performed for barley or wheat interspecific hybrids due to the low rates recombination*.

*(-) QTL mapping was not performed for perennial traits*.

In the grass family, SI is determined by the gametophytic SI system, controlled by the *S* and *Z* loci (Lundqvist, 1957; Cornish et al., 1979). Self-incompatibility occurs when both *S* and *Z* alleles of the pollen and pistil match; a mismatch between *S* and *Z* alleles results in compatibility (Baumann et al., 2000). In the self-incompatible species *Lolium perenne* (2*n* = 2*x* = 14), the *S* locus was mapped to homoeologous group 1 and was found to encode a domain of unknown function (*DUF*) gene (Shinozuka et al., 2010). Meanwhile, in both *L. perenne* and rye (*Secale cereale*; 2*n* = 2*x* = 14), the *Z* locus was mapped to homoeologous group 2 and shown to encode another domain of unknown function gene, *DUF247*, alongside a closely linked ubiquitin-specific protease (*USP*) (Hackauf and Wehling, 2005; Shinozuka et al., 2010; Manzanares et al., 2016). In intermediate wheatgrass (*T. intermedium*; 2*n* = 6*x* = 42), similar SI mechanisms appear to be involved, whereby assessment of progeny combinations resulting from a polycross led to the identification of *S* and *Z* loci gene candidates (Crain et al., 2020b). Consequently, the knockout of these SI candidate loci could increase the fertility of intermediate wheatgrass, and other perennial grain species, stimulating development of self-compatible varieties (Do Canto et al., 2016). Conversely, outcrossing is associated with greater genetic diversity, whereby the high intraspecific diversity of perennial grain species could deliver greater yield stability and ecosystem services when compared to monocultures (Reiss and Drinkwater, 2018; Kreitzman et al., 2022). Furthermore, the ability to genetically control both self-compatibility and incompatibility would allow optimal management of diversity, potentially prompting creation of hybrid cultivars (Do Canto et al., 2016), as has been achieved in annual rye (*Secale cerale*) to great success (Geiger and Miedaner, 1999).

## Promoting and Identifying Opportunities for Perennial Grains in Agriculture

Grain yields of perennial grain crops undergoing domestication are currently lower compared to their annual counterparts. For example, the average grain yield reported for wheat grown in Europe is 5.8 t ha^−1^ (USDA, 2021), which is significantly greater than the 0.3–1.5 t ha^−1^ reported for intermediate wheatgrass (Duchene et al., 2019). When considering this transition, yield should not be the defining characteristic concerning perennial grain crop choice and adoption. For example, growth of lower yielding perennial grain crops could be a great alternative to their higher yielding annual counterparts in areas where their true yield potential is unrealizable. These “yield-gap” scenarios may be associated with soil degradation, strong disease pressure, or low commodity prices, combined with high input costs making production uneconomic, and could complement existing agricultural practice. Therefore, the reduction in input costs, the long-term environmental benefits, and potential dual-use options associated with perennial grain crops, including intermediate wheatgrass, could offset their lower productivity, for which economic analysis has begun (Law et al., 2022).

Regarding intermediate wheatgrass, a survey of growers in the USA revealed cultural acceptance of these ideas. When questioned about their motivations to trial intermediate wheatgrass, growers indicated that their primary areas of interest were improved soil health and microbial communities, reduced tillage requirement, the value of the crop in intercropping and weed suppression, and general interest in the value of perennials on-farm (Lanker et al., 2019; Meijer, 2020). Additionally, following grain harvest regrowth of leaves and stems may be used for grazing livestock, forage, hay, or bedding, or as biomass for biofuel production, potentially increasing the economic return, while the crop's extensive root system would improve soil structure (Ryan et al., 2018; Law et al., 2022).

When considering perennial grain crop adoption, it need not be an either–or situation. The cultivation of perennial grains could complement existing farming practices to aid ecological intensification, supporting the transition from high input intensification that has supported yields of modern, conventionally grown, annual grain crops (Igbozurike, 1978; Bommarco et al., 2013; Yahya et al., 2017). Natural ecosystems are characterized by spatial diversity, i.e., different species grow in the same plot. In annual cropping systems temporal diversity is common, with annual crop rotation using a range of species helping suppress many common pests (Jalli et al., 2021). In perennial systems, intra and interspecific diversity must be deployed in space, through polycultures, for effective pest regulation (Cox et al., 2005). Polyculture, or intercropping—the process of growing two or more crops alongside one another—has been shown to increase stand structural complexity and floristic diversity (Igbozurike, 1978; Yahya et al., 2017; Bybee-Finley and Ryan, 2018; Hirschfeld and Van Acker, 2019).

Compared to monocultures, polycultures are associated with significant environmental benefits, including reduced nitrate leaching, and suppression of weeds, pests, and diseases (Hauggaard-Nielsen et al., 2001, 2003; Thorsted et al., 2006; Picasso et al., 2008). Polycultures are also associated with agronomic improvement, including the increased grain yield and quality reported for cereal–legume intercrops under low-nitrogen conditions (Jensen, 1996; Bedoussac et al., 2015; Crews et al., 2022). Incorporation of perennial grains into polycultures may be additionally advantageous (Weißhuhn et al., 2017), through increasing soil carbon sequestration (Paustian et al., 2016; Sprunger et al., 2020). Further research is required to identify optimal planting densities and species mixes, as recently investigated for intermediate wheatgrass–alfalfa mixes (Jungers et al., 2017; Li S. et al., 2020). In annual cereal–legume polycultures, the legume typically contributes very little biologically fixed nitrogen to the cereal, whereas the cereals in perennial polycultures benefit from the accumulation of leguminous fixed nitrogen over time, increasing yield stability and soil fertility (Crews et al., 2022). Therefore, a future research target could be the identification of leguminous perennial species for intercropping with perennial grains to gain maximal environmental benefit, and, potentially, greater economic return through delivering two harvestable crops for human consumption (Schlautman et al., 2018).

## Elucidating the Genetic and Physiological Basis of Perennialism

Perennialism is a highly complex trait, unlikely to be conferred by a single gene. Ancestral state reconstructions using phylogenetic approaches demonstrate that annual species are generally derived from perennial ancestors, often *via* multiple independent events within a genus (Friedman and Rubin, 2015; Friedman, 2020). Such events can be observed in phylogenetic trees of many plant families, whereby annual species occur within clades derived from a perennial ancestor. In barley, the divergence of life history strategies occurred around 6 million years ago, giving rise to annual *H. vulgare* and perennial *H. bulbosum* (Blattner, 2006), with similar perennial relatives existing for many annual crop species (Table 1; Liston and Wheeler, 1994; Albach et al., 2004; Frajman and Schönswetter, 2011; Li et al., 2022). Together, this indicates that “annuality” is derived from the perennial life form, independently evolving multiple times. Further supporting this hypothesis is the observation that genes contributing to perennial traits, such as rhizome formation, are dominant, with loss-of-function mutations of such genes commonly found in annuals (Hu et al., 2003).

**Figure 3:**
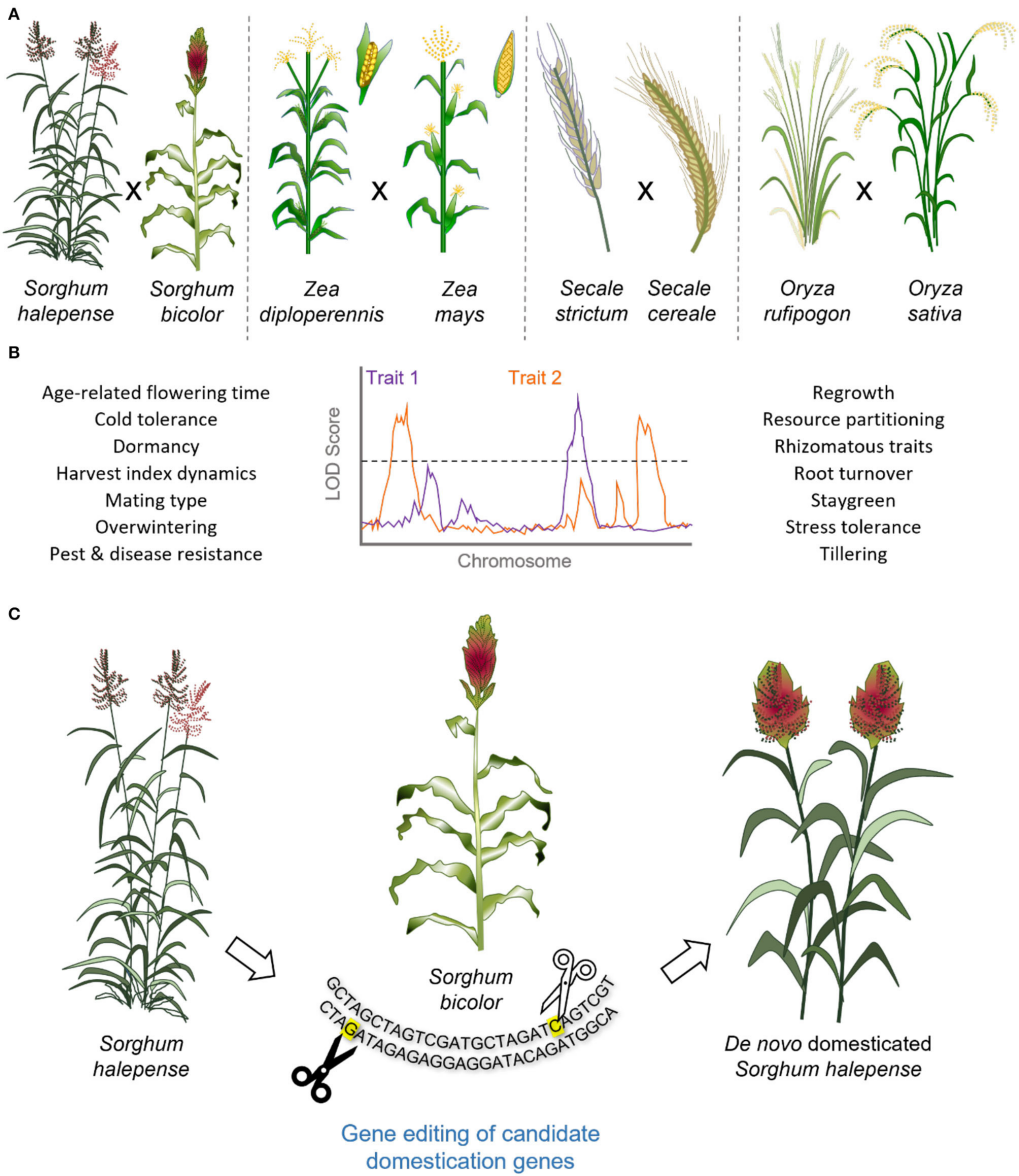
The genetics of perennialism and *de novo* domestication of perennial grain crops. **(A)** Interspecific crosses between perennial relatives and their annual counterparts have been attempted to facilitate QTL analysis and genetic mapping of traits associated with perennialism. **(B)** Perennialism is a complex trait, associated with distinct physiological characteristics and responses to the environmental cues, stresses, diseases, and pests. **(C)** Accelerated domestication of perennial grasses through gene editing of domestication related genes, as identified in their annual relatives, could be used in *de novo* domestication and generation of high-yielding perennial grain crops, illustrated for *Sorghum sp*. The QTL plot reported is for the illustrative purposes only and emphasizes the polygenic nature of perennialism.

Significant effort has been made to elucidate the genetics of perennialism in multiple grain crop species. One approach has been to cross annual grain crops with their perennial relatives to create segregating populations for performance of quantitative trait locus (QTL) mapping (Figures 3A,B; Table 1). The development of such interspecific hybrids is not without its difficulties. For example, in rice, there are multiple reproductive barriers that result in hybrid sterility, including the presence of lethality genes (Li J. et al., 2020). In rye, crossing of annual rye (*S. cereale*) with perennial *S. strictum* resulted in multivalent formation during meiosis and chromosomal translocations, reducing fertility (Gruner and Miedaner, 2021). Similarly, reductions in fertility were observed when attempting to introduce perennial traits from perennial *Thinopyrum* and *Agropyron* species into *T. aestivum*, with crossing efficiencies as low as 18% reported (Armstrong, 1936). Such observations likely relate to the genetic instability associated with ploidy variation as genomic composition of the *Thinopyrum* genus, ranging from diploid (2*n* = 2*x* = 14) to autoallodecaploid (2*n* = 10*x* = 70), with aneuploid and amphidiploid events routinely reported (Nemeth et al., 2015; Cui et al., 2018). Generation of novel interspecific hybrids can also be limited by low recombination frequency, as seen for crosses between barley and *H. bulbosum* (Johnston et al., 2009), curtailing trait mapping.

Crosses between annual *O. sativa* and perennial *O. longistaminata* species have led to the identification of multiple QTLs relating to rhizome formation (Figure 3; Table 1), for which two dominant QTLs, *Rhz2* and *Rhz3*, were originally identified (Hu et al., 2003) and subsequently an additional five major loci (Fan et al., 2020). Similarly, in rye, QTL analysis performed for an interspecific *S. cereale* (annual) *x S. strictum* (perennial) population identified five QTLs accounting for 74% of variation in perennation (Gruner and Miedaner, 2021). Here, perenniality was assessed according to the number of new tillers emerging from initial shoots from the stubble (Figure 3; Gruner and Miedaner, 2021). Interestingly, perenniality within this *S. cereale x S. cristatum* population was found to be influenced by the environment, particularly planting density (Gruner and Miedaner, 2021).

If perenniality in rye is similar to that of maize (*Zea sp*.), such a narrow definition of the perennial trait may not account for wider physiological differences associated with the differences in life cycles. For example, genetic analysis conducted using populations derived from crossing annual *Z. mays* with its perennial relative *Z. diploperennis* led to identification of QTLs associated with tillering (Westerbergh and Doebley, 2004), staygreen (Coatney, 2015), and perennial regrowth (Ma et al., 2019; Swentowsky et al., 2021) traits (Figure 3B). Furthermore, the variation associated with each QTL varies, totaling 27% for a single QTL reported for staygreen (Coatney, 2015), while in combination, the loci *reg1, reg2*, and *reg3* accounted for nearly all variation in perennial regrowth (Swentowsky et al., 2021), re-emphasizing perenniality is a multi-genic trait.

Perception of daylength likely varies between annual and perennial plants. A certain combination of daylength and temperature ensures adequate vernalization of autumn-sown annual cereals (Figure 1). Subsequently, the vegetative-to-floral transition is triggered in response to increasing daylengths. In annual plants, this event occurs once, but in perennial plants such light cues are repeatedly perceived and responded to, and at least three candidate genes, including MADS-box *SEPELLATA2*, have been identified in *Miscanthus* and switchgrass *(Panicum virgatum)* (Jensen et al., 2021). In contrast, within the dicot lineage, a single gene, *PERPETUAL FLOWERING1 (PEP1)*, has been identified as the primary regulator in the perennial species *Arabis alpina* (Wang et al., 2009).

Increased stress resilience of perennial species enables them to tolerate late-seasonal stresses (Westerbergh et al., 2018; Lundgren and Des Marais, 2020), for which annual grain crops have been bred to avoid, primarily through the manipulation of time to flowering and maturation. Therefore, to aid environmental persistence, the genetic basis of cold, heat, drought, and freezing tolerance among perennials needs to be studied. In barley, genome sequencing of perennial *H. bulbosum* and annual *H. vulgare* is facilitating such research. As an alternative to the creation of interspecific annual–perennial crosses, presence and absence variant (PAV) analysis has been conducted between the two species to identify genetic differences (Fuerst et al., 2021). Within the *H. bulbosum* genome, Fuerst et al. (2021) identified a significant enrichment of developmental and disease resistance genes absent among annual *Poaceae* species, alongside those associated with bulb formation relating to starch biosynthesis. These findings, and further interrogation of this PAV dataset, should help identify the genes and processes responsible for perennial-to-annual conversion, making them available for reverse engineering.

Taken together, based on the assumption that annualism is a derived trait (Friedman, 2020), loss of function of just a few genes could, in principle, underly the perennial–annual transition. However, to establish perennialism in an annual species may require the introduction of a much larger number of genes, many of which currently are unknown, and potentially unique to the tertiary gene pools of grain crop species.

## Major Achievements in Introducing Perennialism Into Annual Grain Crops

In breeding, wide hybridization between different species has been successfully used to introgress novel traits (Dwivedi et al., 2008; Kopecký et al., 2022) and create new crops, including triticale (*Triticum* sp. x *Secale* sp.) (Zillinsky, 1973). Using this strategy, crossing of annual grain crop species to their close perennial relatives has been attempted to combine perennial traits with the high-yielding ability associated with annuals (Figure 3A), for which rice is most advanced. Perennial rice was developed through hybridization between annual rice (*Oryza sativa* ssp. *indica*) and the rhizomatous perennial relative, *O. longistaminata* (Tao and Sripichitt, 2000; Sacks et al., 2003). Following backcrossing to annual rice and subsequent breeding, perennial rice cultivars with grain yields comparable to annual rice and capable of persisting for eight harvests are now available (Huang et al., 2018; Zhang et al., 2019; Hu et al., 2022). However, among the major challenges are stability of the perennial trait, fertility among progeny, and maintenance of perennialism during development of high-yielding lines. Although the development of perennial paddy rice may have involved only minor genetic changes, the generation of perennial crops for regions experiencing freezing or drought stress will likely require multiple, unlinked, and currently unknown genes to aid stress tolerance.

Similarly, perennial grain sorghum is being developed through hybridization of annual grain sorghum (*S. bicolor*) with perennial *S. halepense* (Figure 3A). Although hybrid progeny often struggle to survive in cold temperate climates, production of high-yielding perennial varieties could be feasible under warmer conditions (Cox et al., 2018). Moreover, the development of new diploid perennial sorghum lines, which can be readily crossed to locally adapted grain sorghum varieties, could hopefully accelerate development of perennial sorghum throughout the crop's current production range (Cox et al., 2018). Other perennial grains currently under development through wide hybridization are rye (Daly et al., 2022) and perennial wheat (Hayes et al., 2018), with work underway to enhance perennial survival and ensure genetic stability.

## Toward Domestication of Existing Perennial Grain Crops

An alternative strategy to annual-to-perennial conversion is accelerated domestication of existing, low-yielding perennial grain species (Figure 3C; Østerberg et al., 2017), paralleling the repeated selection of major domestication genes and breeding improvement over millennia (Olsen and Wendel, 2013). Previous crop domestication events underpinning the transition from wild to crop species are shown to result from the artificial selection of natural mutations in genes controlling agronomically important traits (Haas et al., 2019). These “domestication genes” influence traits related to improving primary and harvestable grain yield for farmers, regulating plant structure, dormancy, and spike morphology, including threshability, harvestability, and lodging resistance (Olsen and Wendel, 2013; Haas et al., 2019).

Presently, plant breeding has been used to increase productive capacity of perennial species, in conjunction with the optimization of agronomic practices (Crews and Cattani, 2018; Crews et al., 2018). One such wild perennial grain is intermediate wheatgrass (*T. intermedium*), a perennial grass that has mostly been utilized for forage but is now being developed for use as a perennial grain crop through *de novo* domestication. Currently, the crop is produced at a small scale in the USA, sold under the trade name Kernza®, and incorporated into specialty products (DeHaan and Ismail, 2017). The yield potential of intermediate wheatgrass is currently half that of wheat grown under similar conditions (Culman et al., 2013), making the development of high yielding genotypes a key priority. Therefore, breeding programs have been initiated with the aim of increasing both agronomic performance and grain yield (DeHaan et al., 2005, 2018, 2020), for which progress is being greatly accelerated through the application of genomic selection at the seedling stage (Crain et al., 2021a,b).

Other seed-bearing species that are currently targets for accelerated domestication include the perennial sunflower relative *Silphium integrifolium* (Van Tassel et al., 2017), perennial flax (*Linum* sp.) (Tork et al., 2019), and various perennial legume species (Schlautman et al., 2018). Few studies have examined the biochemical and genetic differences underpinning differences in growth, development, and physiology of annual and perennial grain species. With respect to our common domesticated crops, such as wheat, rice, maize, and barley, such knowledge could aid breeding and genetic improvement. However, based on the fact that the domestication of wild annual relatives of barley and wheat began 10,000 years ago, there are concerns that domesticating perennial grain crops may take too long (Cassman and Connor, 2022).

Crews and Cattani (2018) proposed the deployment of two major approaches for the development of new perennial grain crops: *de novo* or direct domestication and wide hybridization (Figure 3). Applications of the *de novo* domestication strategy to intermediate wheatgrass, *H. bulbosum*, and other potential perennial crops (Westerbergh et al., 2018; DeHaan et al., 2020) can learn from previous domestication events, for which the genes regulating traits including brittle rachis, threshability, plant height, grain protein content, and seed size have been identified. As the *de novo* domestication approach for intermediate wheatgrass progresses, nested association mapping population and genome-wide association studies, using genotyping by sequencing, are facilitating QTL analysis and refinement of genomic selection practices (Zhang et al., 2016, 2017; Larson et al., 2019; Crain et al., 2020a; Altendorf et al., 2021b,c, 2022; Figure 3).

Conversely, the wide hybridization approach is based on crossing annual cultivars and perennial wild relatives with the goal of obtaining high-yielding perennials. However, using crosses and selection for perennial crop improvement is complicated due to the complex genetics, polyploid nature, heterozygosity, self-incompatibility, and long generation times of these crops (Schaart et al., 2016). Fortunately, high-quality genome sequences of perennial plants, improved molecular screening methods, and new breeding technologies support the development of highly productive perennial crops capable of fulfilling demands associated with more sustainable agricultural practices (DeHaan et al., 2020).

## Considerations When Developing Perennial Grain Crop Breeding Programs

When screening diversity panels, mutants, or new hybrids for desired characteristics for future exploitation to aid crop improvement, selection accuracy typically depends on the assessment of large populations, potentially spanning a large area. However, phenotyping methods are often subjective and labor-intensive, which may limit both the quantity and quality of data collectable for large-scale breeding programs. High-throughput phenotyping platforms (HTPs) have developed rapidly in the last few years, utilizing a wide range of ground-based sensors or airborne platforms, including drones, for the collection of phenotypic and climatic data (Araus and Cairns, 2014; Crossa et al., 2021). Collected data are later analyzed using image-processing pipelines (Rahaman et al., 2015) and are increasingly integrated and used successfully in the breeding of major food crops (Yang et al., 2020). In perennials, such technology has gained significant traction regarding breeding of forage crop *L. perenne* (Yates et al., 2019; Jayasinghe et al., 2021), and could similarly aid perennial grain crop development. For example, to better assess perennialization and yield stability traits of perennial grain crops, repeated phenotyping of the same plant stands over multiple years would be highly desirable (Figure 2D). In intermediate wheatgrass, genomic selection is currently performed based on phenotypic data over three or more years (Figure 2C; Crain et al., 2021a,b), allowing for rapid progress and improvement of traits requiring multi-year phenotyping.

The desire for multi-year phenotyping when breeding perennial crops has significant implications for breeding program size (Figure 2D). In annual crop breeding, only around 10–20% of lines are selected per season for continuation, with the rest discarded, making way for new material. However, phenotyping, and recurrent annual selection of each breeding cycle over a 3–4-year period would require multiple breeding cycles to be phenotyped simultaneously, with the program running the risk of exponential growth due to maintenance of overlapping cycles, particularly if the cycles are selected concurrently. Consequently, this would necessitate a major increase in the amount of phenotypic assessment performed and associated investment.

When breeding perennial grain crops, attention must be paid to the cropping practice to be used. Spaced plants, polycultures, and monocultures provide different competition environments for which the response of different perennial grain crop candidates should be tested to design optimum production systems. In this regard, classical quantitative genetic parameters and QTL identification for biomass, morphology, and forage nutritive value have been investigated (Mortenson et al., 2019; Altendorf et al., 2021a).

Upon selection of potential perennial grain crop candidates and identification of desirable genetic variants, subsequent breeding steps are likely required for trait introgression, likely relating to the removal of undesired background mutations, disruption of linkage drag, or making target loci homozygous. The long generation time of perennials makes this process very time-consuming, but just as has been performed in multiple annual crops, “speed breeding” methodologies could be a useful tool to accelerate breeding and research programs (Watson et al., 2018). For example, through the optimization of photoperiod, temperature, plant density, and watering regime, speed breeding protocols can significantly shorten generation times, as exemplified in spring wheat, in which use of speed breeding techniques can achieve six generations per year instead of the two to three under glasshouse conditions (Watson et al., 2018). Speed breeding is particularly promising when paired with genomic selection models, since models developed based on field performance for many years could be implemented in a rapid-cycling speed breeding program. Moreover, HTP approaches could be further developed to screen germplasm in resource-limited environments and for pathogen and pest resistance (Ghanem et al., 2015).

## Using Induced Random Mutagenesis to Unlock the Potential of Perennial Grains

While the genetic regulation of perenniality is complex, the introduction and identification of novel alleles for domestication and agronomic trait improvement, e.g., high yield, within existing perennial germplasm should aid perennial grain crop development. To unlock the potential of perennial grains, it is essential to introduce beneficial alleles for these traits, be they naturally occurring, induced, or edited (Fernie and Yan, 2019; DeHaan et al., 2020). Based on reverse genetic techniques, such beneficial genetic variants can be identified using the recently developed method FIND-IT (fast identification of nucleotide variants by digital PCR) (Knudsen et al., 2021). FIND-IT utilizes the high sensitivity of droplet digital PCR to identify chemically induced SNPs (Single Nucleotide Polymorphism) present in large mutant populations, including knockout and promoter mutations, in addition to non-synonymous codon mutations and those in miRNA binding sites (Knudsen et al., 2021). Compared to conventional TILLING (targeting induced local lesions in genomes) approaches (Barkley and Wang, 2008), the FIND-IT method can handle 50–100 times larger population sizes, greatly increasing the likelihood of finding specific mutants. The number of background mutations introduced by the chemical treatment for the FIND-IT technique is similar to that introduced by tissue culture techniques associated with transformation systems needed for gene editing technologies (Graham et al., 2020) and is easily tolerated in the downstream breeding pipeline. Furthermore, given that most chemically induced mutations are thought to be recessive (Parry et al., 2009; Rakszegi et al., 2010), FIND-IT could be used to identify genetic variants that may otherwise be lost during phenotypic screens, selection, and from breeding programs.

As our knowledge of target perennial crops improves, future introgression of beneficial variants, alongside identified QTLs, could be used in the deployment of marker-assisted selection for trait improvement. In intermediate wheatgrass, such efforts may be challenging due to the polyploid, outcrossing, and highly heterozygous nature of this species, for which other promising perennials, including perennial legumes and sunflower (*Helianthus*) species share commonalities (Cox et al., 2006; Kantar et al., 2014; Crews and Cattani, 2018). Therefore, the use of traditional introgression techniques conducted *via* line-breeding approaches, as conducted for annual species, may be inappropriate and require rethinking. Furthermore, when breeding perennials, phenotyping over multiple seasons is required to assess yield stability and long-term climate resilience, necessitating maintenance of plant stands over several years, in sharp contrast to annuals.

## Using Precision Mutagenesis by Genome Editing to Domesticate Perennial Grains

The development of genome editing techniques has enabled the introduction of precise and predictable mutagenesis of domestication genes and therefore may be used for accelerated domestication of wild perennial grain crops (Figure 3C). The recent results for wild annuals such as groundcherry (*Physalis pruinosa*) (Lemmon et al., 2018), ancestral tomato (*Lycopersicon pimpinellifolium*) (Li et al., 2018; Zsögön et al., 2018), and wild allotetraploid rice (*Oryza alta*) (Yu et al., 2021) suggest previous domestication of wild plants could be mimicked through mutagenesis of fewer than 10 genes.

Regeneration is the prerequisite of genome editing in plants. Unfortunately, most perennial grain crop candidates are regeneration recalcitrant (McCown, 2000), with low regeneration efficiencies representing a major bottleneck to genome editing. To improve plant regeneration efficiency, boosters, a type of developmental regulator, can be used. For example, *WUSCHEL* (*WUS*) and *BABY BOOM* (*BBM*) have been shown to improve the regeneration frequency of a variety of transformation recalcitrant annual grain crops, including maize, sorghum, and *indica* rice (*O. sativa* ssp. *indica*) (Lowe et al., 2016). Optimized promoters have been used to drive the spatiotemporal expression of *WUS* and *BBM*, preventing their adverse effects (Lowe et al., 2018). In addition, growth-regulating factors (GRFs), GRF-interacting factors (GIFS), and GRF–GIF chimeras have been used to boost the regeneration of annual plant species (Debernardi et al., 2020; Kong et al., 2020; Luo and Palmgren, 2021), but their use in perennials requires testing. Recently, *TaWOX5* was proven to improve wheat regeneration, reducing genotypic dependency of plant transformation protocols (Wang et al., 2022). Due to the genetic similarity of wheat to both *T. intermedium* and *H. bulbosum*, identification and use of *TaWOX5* orthologs, and similar boosters, may improve perennial crop transformation. Gene editing through *de novo* induction of meristems (Maher et al., 2020) may, in future, allow for the direct transformation of soil-grown perennial species, which would help bypass the need for time-consuming, and often inefficient transformation techniques based on cultured cells.

## Understanding the Transcriptional Landscape as a Short-Cut to Trait Improvement

Many promising perennial grain crops are polyploid, including intermediate wheatgrass, *H. bulbosum* (Westerbergh et al., 2018) and *S. halepense* (Paterson et al., 2020). Polyploidy results in multiple gene copies, which can originate from a single (autopolyploid) or multiple (allopolyploid) species. In maize, and particularly hexaploid wheat, significant transcriptional dynamism has been reported, whereby not all homoeologs contribute equally to gene expression (Ramírez-González et al., 2018; Wang et al., 2018). For example, in wheat, patterns of unbalanced gene expression have been reported relating to the A, B, and D subgenomes, with instances of subgenome dominance, balanced, or tissue-specific homoeologous expression (Ramírez-González et al., 2018). With advances in genomics, it may be possible to test if such transcriptional dynamism exists in perennial polyploids, opening the possibility of modulating homoeologous allelic variation through targeting of dominant gene copies for improvement of quantitative traits.

More widely, targeting of microRNAs (miRNAs) could also be used to modulate the expression of both homologous and homoeologous gene copies. MicroRNAs are small molecules that target specific mRNA targets based on their sequence complementarity. Through miRNA binding, mRNAs are targeted for degradation or repression of translation, contributing to the regulation of developmental and physiological processes. In rice, the disruption of miR396 recognition sites in *OsGRF4* (*GROWTH REGULATING FACTOR4*) results in increased levels of *GRF4* transcripts, contributing to the enlarged grain size of transgenic rice lines (Li et al., 2016). In polyploids, targeting of miRNA binding sites either through mutagenesis or CRISPR could be a way to obtain gain-of-function mutations. In addition, the similarity between homoeologous gene copies would alleviate the need to identify mutations in all subgenomes, reducing the time required to introduce a given phenotype into existing germplasm.

## *Hordeum bulbosum*: A Case Study of a Perennial Barley Candidate Species

As an example of the challenges related to domestication of a perennial grain crop, *H. bulbosum* will be briefly outlined, while those related to *T. intermedium* (Kernza®) have been reported elsewhere (DeHaan et al., 2020).

For the major crop species *H. vulgare*, multiple perennial relatives exist within the *Hordeum* genus. Of these, *H. bulbosum* is the closest relative, and was selected as the candidate species for development of perennial barley following in-field evaluation of 17 perennial *Hordeum* species in Uppsala, Central Sweden from 2013 to 2016 (Westerbergh et al., 2018). Perenniality of *H. bulbosum* is made possible by the meristems in buds located below its bulbous storage organ at the base of the tillers, enabling the plant to persist through a dry summer and cold winter in a dormant state (Westerbergh et al., 2018). Furthermore, field evaluation of 90 diploid and tetraploid *H. bulbosum* accessions, totaling ~700 genotypes, conducted in Central Sweden from 2017 to 2021 also revealed significant genetic and phenotypic diversity (Westerbergh, in prep). Accessions evaluated were primarily sourced through the Nordic Genetic Resource Centre (NordGen) and selected to represent the species' original distribution from regions around the Mediterranean Sea to Central Asia. The best performing genotypes have been selected, with offspring used for breeding and development of perennial barley.

Could the wide hybridization or accelerated domestication strategies be used successfully for development of perennial barley? Following crosses between barley and *H. bulbosum*, the elimination of *H. bulbosum* chromosomes is common, resulting in barley haploids (Gernand et al., 2006) with few viable hybrids available for further crosses. Nevertheless, under certain conditions introgression lines between barley and *H. bulbosum* have been generated, carrying single *H. bulbosum* segments at terminal chromosomal ends in a barley background (Pickering, 1991; Johnston et al., 2009; Wendler et al., 2015). These introgression lines did, however, not show perennial winter survival when cultivated in the field in Central Sweden (Westerbergh et al., in prep). This is likely due to the low recombination frequency and the incomplete transfer of genes for perennial traits from *H. bulbosum* into the barley genome. Therefore, the difficulties associated with obtaining perennial offspring from this annual-perennial interspecific *Hordeum* hybrid make this approach for perennial crop development less attractive to pursue. Conversely, the successful identification of perennial *H. bulbosum* genotypes among wild accessions favors adoption of the *de novo* domestication approach to develop perennial barley, for which knowledge of the close relationship between *H. vulgare* and *H. bulbosum* and their respective genetic resources will assist. Early target traits for introduction include domestication traits. In common with other wild barley relatives, all investigated *H. bulbosum* accessions had two-row spikes and brittle rachis phenotypes in which the rachis breaks at the points where spikelets have developed (Westerbergh et al., 2018), making the non-brittle rachis trait key for *H. bulbosum* domestication.

A high-quality genome sequence is useful for the targeted plant breeding approaches. The genomes of *H. bulbosum* and *H. vulgare* are highly similar (Jakob et al., 2004), which may indicate that the two species encode orthologous copies of the same domestication genes. However, unlike *H. vulgare, H. bulbosum* has both a diploid and an autotetraploid form (2*n* = 2*x* = 14; 2*n* = 4*x* = 28), and is outcrossing (von Bothmer et al., 1995; Komatsuda et al., 2004). Diploid *Hordeum* species have large (~5.5 Gb) genomes, containing highly repetitive and complex regions that make whole-genome sequencing and assembly challenging. However, in 2017, the International Barley Genome Sequencing Consortium (IBSC) used hierarchical shotgun sequencing to successfully assemble and sequence the genome of *H. vulgare* cv. Morex (Beier et al., 2017). In parallel, next-generation sequencing methods were used to produce an extensive pool of molecular markers and SNP information for most of the published *H. vulgare/H. bulbosum* introgression lines. Concurrently, genotyping-by-sequencing was used to create high-density genetic maps of *H. bulbosum*, in addition to a partial *de novo* genome assembly for *H. bulbosum*, for which gene models were predicted (Wendler et al., 2017). Notably, the *H. bulbosum* genome is enriched with developmental and disease-responsive genes absent among annual *Poaceae* species (Fuerst et al., 2021).

For the tetraploid form of *H. bulbosum*, four alleles of a given gene may be present in a single individual. Within larger wild *H. bulbosum* populations, each locus likely harbors many allelic variants. Polyploidy in general therefore makes identification of mutants using phenotypic evaluation difficult, especially for rare phenotypes like non-brittle rachis, or low-frequency, recessive alleles; thus, necessitating the sequencing of all allelic variants to design a working strategy. Novel variation in collections or induced by mutagenesis can be identified by TILLING approaches (Barkley and Wang, 2008) or the FIND-IT method (Knudsen et al., 2021). If a recessive mutation is required to produce a desired phenotype, each allele may need to be mutated followed by breeding and selection to obtain homozygosity at all loci.

The goal of domesticating and breeding *H. bulbosum* is to develop the first perennial alternative to barley to produce sustainable high yields. Through combining multiple methods, including utilization of existing diversity within *H. bulbosum* for beneficial traits affecting harvestable yield, integration of phenotypic and genotypic data for the purposes of phenotypic marker-assisted breeding, and genomic selection this goal should be achievable. Furthermore, chemical mutagenesis or gene-editing can be used to introduce novel genetic variation for modification of traits lacking variation within *H. bulbosum*, e.g., brittle rachis, and will also be useful in targeting of known genes controlling quality and yield to complement this.

## Conclusion

Diversity and perenniality are the key to producing stable and sustainable agroecosystems. The success in obtaining a perennial rice cultivar through traditional breeding practices demonstrates that it is indeed possible to obtain a perennial grain crop with yields comparable to annual grain crops. Among the wild relatives of cereal grains, many species are already perennial and adapted to diverse habitats, for which their great genetic diversity would benefit development of wide-ranging perennial grain crops and cultivars. In addition, knowledge of how perennials allocate resources with respect to vegetative growth and seed yield will be necessary during the breeding process to reliably deliver high grain yields from one year to the next. By applying new breeding techniques such as CRISPR and FIND-IT, important domestication traits can be introduced and the speed of development of perennial grain crops can be substantially increased.

## Data Availability Statement

The original contributions presented in the study are included in the article/supplementary material, further inquiries can be directed to the corresponding author/s.

## Author Contributions

EAC contributed significantly to the review structure, ideation, and creation of figures. EAC and MP jointly performed the editing and proofing. All authors contributed to the article and approved the submitted version.

## Funding

This work was supported by grants from the Carlsberg Foundation (RaisingQuinoa, Project Number CF18-1113, MP; Crops for the Future, Project Number CF20-0352, EAC and ST), the Innovation Fund Denmark (LESSISMORE, MP; DEEPROOTS; HCT, MP, and SK), and the Novo Nordisk Foundation (NovoCrops; Project Number 2019OC53580; MP).

## Conflict of Interest

The authors declare that the research was conducted in the absence of any commercial or financial relationships that could be construed as a potential conflict of interest.

## Publisher's Note

All claims expressed in this article are solely those of the authors and do not necessarily represent those of their affiliated organizations, or those of the publisher, the editors and the reviewers. Any product that may be evaluated in this article, or claim that may be made by its manufacturer, is not guaranteed or endorsed by the publisher.

## References

[B1] Aizen M. A. Harder L. D. (2007). Expanding the limits of the pollen-limitation concept: effects of pollen quantity and quality. Ecology 88, 271–281. 10.1890/06-1017 17479745

[B2] Albach D. C. Martínez-Ortega M. M. Chase M. W. (2004). Veronica: parallel morphological evolution and phylogeography in the Mediterranean. Plant Syst. Evol. 246, 177–194. 10.1007/s00606-004-0148-9

[B3] Altendorf K. R. DeHaan L. R. Anderson J. (2022). Genetic architecture of yield-component traits in the new perennial crop, intermediate wheatgrass. Crop Sci. 62, 880–892. 10.1002/csc2.20716

[B4] Altendorf K. R. DeHaan L. R. Heineck G. C. Zhang X. Anderson J. A. (2021a). Floret site utilization and reproductive tiller number are primary components of grain yield in intermediate wheatgrass spaced plants. Crop Sci. 61, 1073–1088. 10.1002/csc2.20385

[B5] Altendorf K. R. DeHaan L. R. Larson S. R. Anderson J. A. (2021b). QTL for seed shattering and threshability in intermediate wheatgrass align closely with well-studied orthologs from wheat, barley, and rice. Plant Genome 14, e20145. 10.1002/tpg2.20145 34626160 PMC12806861

[B6] Altendorf K. R. Larson S. R. DeHaan L. R Crain J. Neyhart J. . (2021c). Nested association mapping reveals the genetics architecture of spike emergence and anthesis timing in intermediate wheatgrass. G3: Genes Genom. Genet. 11, jkab025. 10.1093/g3journal/jkab0025 33890617 PMC8063084

[B7] Altieri M. A. Nicholls C. I. Henoa A. Lana M. A. (2015). Agroecology and the design of climate change-resilient farming systems. Agron. Sustain. Dev. 35, 869–890. 10.1007/s13593-015-0285-2

[B8] Araus J. L. Cairns J. E. (2014). Field high-throughput phenotyping: the new crop breeding frontier. Trends Plant Sci. 19, 52–61. 10.1016/j.tplants.2013.09.008 24139902

[B9] Armstrong J. M. (1936). Hybridization of Triticum and Agropyron: I. crossing results and description of the first generation hybrids. Can. J. Res. 14c, 16. 10.1139/cjr36c-016

[B10] Asseng S. Ewert F. Martre P. Rötter R. P. Lobell D. B. Cammarano D. . (2015). Rising temperatures reduce global wheat production. Nat. Clim. Change 5, 143–147. 10.1038/nclimate2470

[B11] Audu V. Rasche F. Dimitrova Mårtensson L.-M. Emmerling C. (2022). Perennial cereal grain cultivation: Implication on soil organic matter and related soil microbial parameters. Appl. Soil Ecol. 174, 104414. 10.1016/j.aspsoil.2022.104414

[B12] Barkley N. A. Wang M. L. (2008). Application of TILLING and EcoTILLING as reverse genetic approaches to elucidate the function of genes in plants and animals. Curr. Genomics 9, 212–226. 10.2174/138920208784533656 19452039 PMC2682938

[B13] Batello C. Wade L. Cox S. Pogna N. Bozzini A. Choptiany J. (2013). Perennial Crops for Food Security: Proceedings of the FAO Expert Workshop. Rome: Food and Agriculture Organization of the United Nations (FAO).

[B14] Baumann U. Bian X. Langridge P. (2000). Self-incompatibility in the grasses. Ann. Bot. 85, 203–209. 10.1007/978-3-540-68486-2_13

[B15] Beddington J. R. Asaduzzaman M. Bremauntz F. A. Clark M. E. Guillou M. Jahn M. M. . (2012). Achieving Food Security in the Face of Climate Change: Final Report From the Commission on Sustainable Agriculture and Climate Change. Copenhagen, Denmark: CGIAR Research Program on Climate Change, Agriculture and Food Security (CCAFS).

[B16] Bedoussac L. Journet E. Hauggaard-Nielsen H. Naudin C. Corre-Hellou G. Jensen E. S. . (2015). Ecological principles underlying the increase of productivity achieved by cereal-grain legume intercrops in organic farming. a review. Agron. Sustain. Dev. 35, 911–935. 10.1007/s13593-014-0277-7

[B17] Beier S. Himmelbach A. Colmsee C. Zhang X.-Q. Barrero R. A. Zhang Q. . (2017). Construction of a map-based reference genome sequence for barley, *Hordeum vulgare*. Sci. Data 4, 170044. 10.1038/sdata.2017.44 28448065 PMC5407242

[B18] Berger J. Palta J. Vadez V. (2016). Review: an integrated framework for crop adaptation to dry environments: responses to transient and terminal drought. Plant Sci. 253, 58–67. 10.1016/j.plantsci.2016.09.007 27968997

[B19] Blattner F. R. (2006). Multiple intercontinental dispersals shaped the distribution area of *Hordeum* (Poaceae). New Phytol. 169, 603–614. 10.1111/j.1469-8137.20058.01610.x 16411962

[B20] Bommarco R. Kleijn D. Potts S. G. (2013). Ecological intensification: harnessing ecosystem services for food security. Trends Ecol. Evol. 28, 230–238. 10.1016/j.tree.10.012 23153724

[B21] Borrell A. van Oosterom E. Hammer G. Jordan D. Douglas A. (2003). The physiology of “stay-green” in sorghum, in Proceedings of the 11^th^ Australian Agronomy Conference, Victoria: Geelong.

[B22] Borrell A. K. Mullet J. E. George-Jaeggli B. van Oosterom E. J. Hammer G. L. . (2014). Drought adaptation of stay-green sorghum is associated with canopy development, leaf anatomy, root growth, and water uptake. J. Exp. Bot. 65, 6251–6263. 10.1093/jxb/eru232 25381433 PMC4223986

[B23] Borrill P. Fahy B. Smith A. M. Uauy C. (2015). Wheat grain filling is limited by grain filling capacity rather than the duration of flag leaf photosynthesis: a case study using NAM RNAi Plants. PLoS One 10, e0134947. 10.1371/journal.pone.0134947 26241955 PMC4524614

[B24] Buchanan-Wollaston V. (2007). Senescence in Plants, eLS. Chichester: John Wiley and Sons, Ltd.

[B25] Burnett A. C. Rogers A. Rees M. Osborne C. P. (2016). Carbon source-sink limitations differ between two species with contrasting growing strategies. Plant Cell Environ. 39, 2460–2472. 10.1111/pce.12801 27422294

[B26] Bybee-Finley K. A. Ryan M. R. (2018). Advancing intercropping research and practices in industrialized agricultural landscapes. Agriculture 8, 80. 10.3390/agriculture8060080

[B27] Cassman K. G. Connor D. J. (2022). Progress toward perennial grains for prairies and plains. Outlook Agric. 22, 1–7. 10.1177/00307270211073153

[B28] Chapman E. A. Orford S. Lage J. Griffiths S. (2021). Delaying or delivering: identification of novel NAM-1 alleles that delay senescence to extend wheat grain fil duration. J. Exp. Bot. 72, 7710–7728. 10.1093/jxb/erab368 34405865 PMC8660559

[B29] Chibane N. Caicedo M. Martinez S. Marcet P. Revilla P. Ordás B. (2021). Relationship between delayed leaf senescence (stay-green) and agronomic and physiological characters in maize (*Zea mays* L.). Agronomy 11, 276. 10.3390/agronomy11020276

[B30] Coatney C. (2015). Characterization of perennial traits in hybrids between maize and perennial teosinte. Master's Thesis. University of Georgia.

[B31] Cornish M. A. Hayward M. D. Lawrence M. J. (1979). Self-incompatibility in ryegrass: I. Genetic control in diploid Lolium perenne L. Heredity 43, 95–106. 10.1038/hdy.1979.6329626084

[B32] Cox C. M. Garrett K. A. Bockus W. W. (2005). Meeting the challenge of disease management in perennial grain cropping systems. Renew. Agric. Food Syst. 20, 15–24. 10.1079/RAF200495

[B33] Cox S. Nabukalu P. Paterson A. H. Kong W. Auckland S. Rainville L. . (2018). High proportion of diploid hybrids produced by interspecific diploid x tetraploid *Sorghum* hybridization. Genet. Resour. Crop Evol. 65, 387–390. 10.1007/s10722-017-0580-7

[B34] Cox T. S. Glover J. D. Van Tassel D. L. Cox C. M. DeHaan L. R. (2006). Prospects for developing perennial grain crops. Bioscience 56, 649–659. 10.1641/0006-3568(2006)56[649:PFDPGC]2.0.CO;2

[B35] Crain J. Bajgain P. Anderson J. Zhang X. DeHaan L. Poland J. (2020a). Enhancing crop domestication through genomic selection, a case study of intermediate wheatgrass. Front. Plant Sci. 11, 319. 10.3389/fpls.2020.00319 32265968 PMC7105684

[B36] Crain J. DeHaan L. Poland J. (2021a). Genomic prediction enables rapid selection of high-performing genets in an intermediate wheatgrass breeding program. Plant Genome 14, e20080. 10.1002/tpg2.20080 33660427 PMC12899483

[B37] Crain J. Haghighattalab A. DeHaan L. Poland J. (2021b). Development of whole-genome prediction models to increase the rate of genetic gain in intermediate wheatgrass (*Thinopyrum intermedium*) breeding. Plant Genome 14, e20089. 10.1002/tpg2.20089 33900690 PMC12807048

[B38] Crain J. Larson S. Dorn K. Hagedorn T. DeHaan L. Poland J. (2020b). Sequenced-based paternity analysis to improve breeding and identify self-incompatibility loci in intermediate wheatgrass (*Thinopyrum intermedium*). Theor. Appl. Genet. 133, 3217–3233. 10.1007/s00122-020-03666-1 32785739 PMC7547974

[B39] Crews T. Cattani D. (2018). Strategies, advances, and challenges in breeding perennial grain crops. Sustainability 10, 2192. 10.3390/su10072192

[B40] Crews T. E. Blesh J. Culman S. W. Hayes R. C. Jensen E. S. Mack M. . (2016). Going where no grains have gone before: from early to mid-succession. Agricult. Ecosyst. Environ. 223, 223–238. 10.1016/j.agee.2016.03.012

[B41] Crews T. E. Carton W. Olsson L. (2018). Is the future of agriculture perennial? imperatives and opportunities to reinvent agriculture by shifting from annual monocultures to perennial polycultures. Global Sustain. 1, E11. 10.1017/sus.2018.11

[B42] Crews T. E. Kemp L. Bowden J. H. Murrell E. G. (2022). How the nitrogen economy of a perennial cereal-legume intercrop affects productivity: can synchrony be achieved? Front. Sustain. Food Syst. 6, 755548. 10.3389/fsufs.2022.755548

[B43] Crossa J. Fritsche-Neto R. Montesinos-Lopez O. A. Costa-Neto G. Dreisigacker S. Montesinos-Lopez A. . (2021). The modern plant breeding triangle: optimizing the use of genomics, phenomics, and enviromics data. Front. Plant. Sci. 12, 651480. 10.3389/fpls.2021.651480 33936136 PMC8085545

[B44] Cui L. Ren Y. Murray T. D. Yan W. Guo Q. Niu Y. . (2018). Development of perennial wheat through hybridization between wheat and wheatgrasses: a review. Engineering 4, 507–513. 10.1016/j.eng.2018.07.003

[B45] Culman S. W. Snapp S. S. Ollenburger M. Basso B. DeHaan L. R. (2013). Soil and water quality rapidly responds to the perennial grain Kernza wheatgrass. Agron. J. 105, 735–744. 10.2134/agronj2012.0273

[B46] Daly E. J. Hernandez-Ramirez G. Puurveen D. Ducholke C. Kim K. Oatway L. (2022). Perennial rye as a grain crop in Alberta, Canada: prospects and challenges. Agron. J. 114, 471–489. 10.1002/agj2.20965

[B47] de Souza Luche H. Gonzalez da Silva J. A. Nornberg R. Hawerroth M. C. da Silveira Silveira S. F. da Rosa Caetano V. . (2017). Stay-green character and its contribution in Brazilian wheats. Ciência Rural 47, 583. 10.1590/0103-8478cr20160583

[B48] Debernardi J. M. Tricoli D. M. Ercoli M. F. Hayta S. Ronald P. Palatnik J. F. . (2020). A GRF-GIF chimeric protein improves the regeneration efficiency of transgenic plants. Nat. Biotechnol. 38, 1274–1279. 10.1038/s41587-020-0703-0 33046875 PMC7642171

[B49] DeHaan L. Christians M. Crain J. Poland J. (2018). Development and evolution of an intermediate wheatgrass domestication program. Sustainability 10, 1499. 10.3390/su10051499

[B50] DeHaan L. Larson S. López-Marqués R. L. Wenkel S. Gao C. Palmgren M. (2020). Roadmap for accelerated domestication of an emerging perennial grain crop. Trends Plant Sci. 25, 525–537. 10.1016/j.tplants.2020.02.004 32407693

[B51] DeHaan L. Van Tassel D. Cox T. (2005). Perennial grain crops: a synthesis of ecology and plant breeding. Renew. Agric. Food Syst. 20, 5–14. 10.1079/RAF200496

[B52] DeHaan L. R. Ismail B. P. (2017). Perennial cereals provide ecosystem benefits. Cereal Foods World 62, 278–281. 10.1094/CFW-62-6-027829662249

[B53] DeHaan L. R. Wang S. Larson S. R. Catton D. J. Zhang X. Kantarski T. (2013). Current efforts to develop perennial wheat and domesticate *Thinopyrum intermedium* as a perennial grain, in Perennial Crops for Food Security: Proceedings of the FAO Expert Workshop, eds Batello C. Wade L. Cox S. Pogna N. Bozzini A. Choptiany J.

[B54] Díaz S. (2001). Ecosystem function measurement, terrestrial communities. Encyclopedia Biodiv. 2, 321–344. 10.1016/B0-12-226865-2/00089-4

[B55] Do Canto J. Studer B. Lubberstedt T. (2016). Overcoming self-incompatibility in grasses: a pathway to hybrid breeding. Theor. Appl. Genet. 129, 1815–1829. 10.1007/s00122-016-2775-2 27577253

[B56] Dohleman F. G. Long S. P. (2009). More productive than maize in the Midwest: how does *Miscanthus* do it? Plant Phys. 150, 2104–2115. 10.1104/pp.109.139162 19535474 PMC2719137

[B57] Duchene O. Celette F. Ryan M. DeHaan L. Crews T. David C. (2019). Integrating multipurpose perennial grain crops in Western European farming systems. Agricult. Ecosyst. Environ. 284, 106591. 10.1016/j.agee.2019.106591

[B58] Dwivedi S. L. Upadhyaya H. D. Stalker H. T. Blair M. W. Bertiolo D. J. Nielen S. . (2008). Enhancing crop gene pools with beneficial traits using wild relatives. Plant Breed. Rev. 30, 179–230. 10.1002/9780470380130.ch3

[B59] Fan Z. Wang K. Rao J. Cai Z. Tao L. Fan Y. . (2020). Interactions among multiple quantitative trait loci underlie rhizome development of perennial rice. Front. Plant Sci. 11, 591157. 10.3389/fpls.2020.591157 33281851 PMC7689344

[B60] FAO (2021). World Food and Agriculture—Statistical Yearbook 2020.

[B61] Fernie A. R. Yan J. (2019). De novo domestication: an alternative route toward new crops for the future. Mol. Plant 12, 615–631. 10.1016/j.molp.2019.03.016 30999078

[B62] Frajman B. Schönswetter P. (2011). Giants and dwarfs: molecular phylogenies reveal multiple origins of annual spurges within *Euphorbia* subg. Esula. Mol. Phylogenet. Evol. 61, 413–424. 10.1016/j.ympev.2011.06.011 21708275

[B63] Friedman J. (2020). The evolution of annual and perennial plant life histories: ecological correlates and genetic mechanisms. Annu. Rev. Ecol. Evol. Syst. 51, 461–481. 10.1146/annurev-ecolsys-110218-024638

[B64] Friedman J. Rubin M. J. (2015). All in good time: understanding annual and perennial strategies in plants. Am. J. Bot. 102, 497–499. 10.3732/ajb.1500062 25878083

[B65] Fuerst D. Shermeister B. Mandel T. Hübner S. (2021). Decoupling the molecular regulation of perenniality and flowering in bulbous barley (*Hordeum bulbosum*). BioRxiv. 10.1101/2021.11.11.468190 PMC9840211 36449556

[B66] Garland T. Jr. (2014). Trade-offs. Curr. Biol. 24, R60–R61. 10.1016/j.cub.2013.11.036 24456973

[B67] Geiger H. H. Miedaner T. (1999). Hybrid rye and heterosis, in Genetics and Exploitation of Heterosis in Crops, eds Coors J. G. Pandey S. p. 41.

[B68] Gernand D. Rutten T. Pickering R. Houben A. (2006). Elimination of chromosomes in Hordeum vulgare × H. bulbosum crosses at mitosis and interphase involves micronucleus formation and progressive heterochromatinization. Cytogenet. Genome Res. 114, 169–174. 10.1159/000093334 16825770

[B69] Ghanem M. Marrou H. Sinclair T. (2015). Physiological phenotyping of plants for crop improvement. Trends Plant Sci. 20, 139–144. 10.1016/j.tplants.2014.11.006 25524213

[B70] Glover J. D. Reganold J. P. Bell L. W. Borevitz J. Brummer E. C. Bucker E. . (2010). Increased food and ecosystem security *via* perennial grains. Science 328, 1638–1639. 10.1126/science.1188761 20576874

[B71] González-Paleo L. Ravetta D. A. (2015). Carbon acquisition strategies uncoupled from predictions derived from species life-cycle. Flora 212, 1–9. 10.1016/j.flora.2015.02.004

[B72] Graham N. Patil G. B. Bubeck D. M. Dobert R. C. Glenn K. C. Gutsche A. T. . (2020). Plant genome editing and the relevance of off-target changes. Plant Physiol. 183, 1453–1471. 10.1104/pp.19.01194 32457089 PMC7401131

[B73] Gregersen P. L. Culetic A. Boschian L. Krupinska K. (2013). Plant senescence and crop productivity. Plant Mol. Biol. 82, 603–622. 10.1007/s11103-013-0013-8 23354836

[B74] Gruner P. Miedaner T. (2021). Perennial rye: genetics of perenniality and limited fertility. Plants 10, 1210. 10.3390/plants10061210 34198672 PMC8232189

[B75] Guo L. Plunkert M. Luo X. Liu Z. (2021). Developmental regulation of stolon and rhizome. Curr. Opin. Plant Biol. 59, 101970. 10.1016/j.pbi.2020.10.003 33296747

[B76] Haas M. Schreiber M. Mascher M. (2019). Domestication and crop evolution of wheat and barley: genes, genomics, and future directions. J. Integr. Plant Biol. 61, 204–225. 10.1111/jipb.12737 30414305

[B77] Habyarimana E. De Franceschi P. Ercisli S. Shehzad F. S. Dall'Agata M. (2020). Genome-wide association study for biomass related traits in a panel of *Sorghum bicolor* and *S. bicolor x S. halepense populations*. Front. Plant Sci. 11, 551305. 10.3389/fpls.2020.551305 33281836 PMC7688983

[B78] Hackauf B. Wehling P. (2005). Approaching the self-incompatibility locus Z in rye (*Secale cereale* L.) *via* comparative genetics. Theor. Appl. Genet. 110, 832–845. 10.1007/s00122-004-1869-4 15717193

[B79] Harlan J. R. DeWet J. M. J. Price E. G. (1973). Comparative evolution of cereals. Evolution 27, 311–325. 10.1111/j.1558-5646.1973.tb00676.x 28564784

[B80] Hauggaard-Nielsen H. Ambus P. Jensen E. S. (2001). Interspecific competition, N use and interference with weeds in pea-barley intercropping. Field Crops Res. 70, 101–109. 10.1016/S0378-4290(01)00126-5

[B81] Hauggaard-Nielsen H. Ambus P. Jensen E. S. (2003). The comparison of nitrogen use and leaching in sole cropped vs. intercropped pea and barley. Nutr. Cycling Agroecosyst. 65, 289–300. 10.1023/A:1022612528161

[B82] Hayes R. C. Wang S. Newell M. T. Turner K. Larsen J. Gazza L. . (2018). The performance of early-generation perennial winter cereals at 21 sites across four continents. Sustainability 10, 1124. 10.3390/su10041124

[B83] Hirschfeld S. Van Acker R. (2019). Permaculture farmers consistently cultivate perennials, crop diversity, landscape heterogeneity and nature conservation. Renew. Agric. Food Syst. 35, 342–351. 10.1017/S1742170519000012

[B84] Hu F. Wang D. Zhao X. Zhang T. Sun H. Zhu L. . (2011). Identification of rhizome-specific genes by genome-wide differential expression analysis in *Oryza longistaminata*. BMC Plant Biol. 11, 18. 10.1186/1471-2229-11-18 21261937 PMC3036607

[B85] Hu F. Zhang S. Huang G. Zhang Y. Lv X. Wan K. . (2022). Perennial rice improves farmer livelihood and ecosystem security. ResearchSquare. 22, 77. 10.21203/rs.3.rs-1302277/v1

[B86] Hu F. Y. Tao D. Y. Sacks E. Fu B. Y. Xu J. Li J. . (2003). Convergent evolution of perenniality in rice and sorghum. Proc. Natl. Acad. Sci. U.S.A. 100, 4050–4054. 10.1073/pnas.0630531100 12642667 PMC153046

[B87] Huang G. Qin S. Zhang S. Cai X. Wu S. Dao J. . (2018). Performance, economics and potential impact of perennial rice PR23 relative to annual rice cultivars at multiple locations in Yunnan orovince of China. Sustainability 10, 1086. 10.3390/su10041086

[B88] Hunter M. C. Sheaffer C. C. Culman S. W. Jungers J. M. (2020). Effects of defoliation and row spacing on intermediate wheatgrass I: grain production. Agron. J. 112, 1748–1763, 10.1002/agj2.20128

[B89] Igbozurike U. M. (1978). Polyculture and monoculture: contrast and analysis. GeoJournal 2, 443–449

[B90] IPCC (2019). Climate Change and Land: an IPCC special report on climate change, desertification, land degradation, sustainable land management, food security, and greenhouse gas fluxes in terrestrial ecosystems, eds P. R. Shukla, J. Skea, E. Calvo Buendia, V. Masson-Delmotte, H.-O. Pörtner, D. C. Roberts, P. Zhai, R. Slade, S. Connors, R. van Diemen, M. Ferrat, E. Haughey, S. Luz, S. Neogi, M. Pathak, J. Petzold, J. Portugal Pereira, P. Vyas, E. Huntley, K. Kissick, M. Belkacemi, J. Malley. In press. Available online at: https://www.ipcc.ch/site/assets/uploads/2019/11/SRCCL-Full-Report-Compiled-191128.pdf

[B91] Jaikumar N. S. Snapp S. S. Sharkley T. D. (2016). Older *Thinopyrum* (Poaceae) plants exhibit superior photosynthetic tolerance to cold stress and greater increases in two photosynthetic enzymes under freezing stress compared with young plants. J. Exp. Bot. 67, 4743–4753. 10.1093/jxb/erw253 27401911 PMC4973744

[B92] Jakob S. S. Meister A. Blattner F. R. (2004). The considerable genome size variation of *Hordeum* species (Poaceae) is linked to phylogeny, life form, ecology, and speciation rates. Mol. Biol. Evol. 21, 860–869. 10.1093/molbev/msh092 15014163

[B93] Jalli M. Huusela E. Jalli H. Kauppi K. Niemi M. Himanen S. . (2021). Effects of crop rotation on spring wheat yield and pest occurrence in different tillage systems: a multi-year experiment in Finnish growing conditions. Front. Sustain. Food Syst. 5, 647335. 10.3389/fsufs.2021.647335

[B94] Jayasinghe C. Badenhorst P. Jacobs J. Spandenberg G. Smith K. (2021). Image-based high-throughput phenotyping for the estimation of persistence of perennial ryegrass (*Lolium perenne* L.)—a review. Grass Forage Sci. 76, 321–339. 10.1111/gfs.12520

[B95] Jensen E. Shafiei R. Ma X. Serba D. D Smith D. P. . (2021). Linkage mapping evidence for a syntenic QTL associated with flowering time in perennial C4 rhizomatous grasses Miscanthus and switchgrass. GCB Bioenergy 13, 98–111. 10.1111/gcbb.12755 33381230 PMC7756372

[B96] Jensen E. S. (1996). Grain yield, symbiotic N_2_ fixation and interspecific competition for inorganic N in pea-barley intercrops. Plant Soil 182, 25–38. 10.1007/BF00010992

[B97] Johnston P. A. Timmerman-Vaughan G. M. Farnden K. J. Pickering R. (2009). Marker development and characterization of *Hordeum bulbosum* introgression lines: a resource for barley improvement. Theor. Appl. Genet. 118, 1429–1437. 10.1007/s00122-009-0992-7 19263032

[B98] Jump A. S. Peñuelas J. (2005). Running to stand still: adaptation and the response of plants to rapid climate change. Ecol. Lett. 8, 1010–1020. 10.1111/j.1461-0248.2005.00796.x 34517682

[B99] Jungers J. M. DeHaan L. R. Betts K. J. Sheaffer C. C. Wyse D. L. (2017). Intermediate wheatgrass grain and forage yield responses to nitrogen fertilization. Agron. J. 109, 462–472. 10.2134/agronj2016.07.0438

[B100] Kantar M. B. Baute G. J. Bock D. G. Rieseberg L. H. (2014). Genomic variation in *Helianthus*: learning from the past and looking to the future. Brief. Funct. Genomics 13, 328–340. 10.1093/bfgp/elu004 24590235

[B101] Kantar M. B. Tyl C. E. Dorn K. M. Zhang X. Jungers J. M. Kaser J. M. . (2016). Perennial grain and oilseed crops. Annu. Rev. Plant Biol. 29, 703–729. 10.1146/annurev-arplant-043015-112311 26789233

[B102] Knudsen S. Wendt T. Dockter C. Thomsen H. C. Rasmussen M. Jørgensen M. E. . (2021). FIND-IT: Ultrafast mining of genome diversity. BioRxiv [Preprint]. 10.1101/2021.05.20.444969

[B103] Komatsuda T. Maxim P. Senthil N. Mano Y. (2004). High-density AFLP map of *nonbrittle rachis 1* (*btr1*) and *2* (*btr2*) genes in barley (*Hordeum vulgare* L.). Theor. Appl. Genet. 109, 986–995. 10.1007/s00122-004-1710-0 15490100

[B104] Kong J. Martin-Ortigosa S. Finer J. Orchard N. Gunadi A. Batts L. A. . (2020). Overexpression of the transcription factor GROWTH-REGULATING FACTOR5 improves transformation of dicot and monocot species. Front. Plant Sci. 11, 572319. 10.3389/fpls.2020.572319 33154762 PMC7585916

[B105] Kong W. Nabukalu P. Cox T. S. Goff V. H. Robertson J. S. Pierce G. J. . (2021). Quantitative trait mapping of plant architecture in two BC1F2 populations of *Sorghum bicolor* x *S. halepense* and comparisons to two other sorghum populations. Theor. Appl. Genet. 134, 1185–1200. 10.1007/s00122-020-03763-1 33423085

[B106] Kopecký D. Martín A. Smýkal P. (2022). Interspecific hybridization and plant breeding: from historical retrospective through work of Mendel to current crops. Czech J. Genet. Plant Breed. 20, 1–14. 10.17221/19/2022-CJGPB

[B107] Kreitzman M. Eyster H. Mitchel M. Czajewska A. Keeley K. Smukler S. . (2022). Woody perennial polycultures in the U.S. Midwest enhance biodiversity and ecosystem functions. Ecosphere 13, e03890. 10.1002/ecs2.3890

[B108] Kreitzman M. Toensmeier E. Chan K. Smukler S. Ramankutty N. (2020). Perennial staple crops: yields, distribution, and nutrition in the global food system. Front. Sustain. Food Syst. 4, 588988 10.3389/fsufs.2020.588988

[B109] Lanker M. Bell M. Picasso V. D. (2019). Farmer perspectives and experiences introducing the novel perennial grain Kernza intermediate wheatgrass in the US Midwest. Renew. Agric. Food Syst. 35, 653–662. 10.1017/S174210519000310

[B110] Larson S. DeHaan L. Poland J. Zhang X. Dorn K. Kantarski T. . (2019). Genome mapping of quantitative trait loci (QTL) controlling domestication traits of intermediate wheatgrass (*Thinopyrum intermedium*). Theor. Appl. Genet. 132, 2325–2351. 10.1007/s00122-019-03357-6 31172227

[B111] Law E. Pelzer C. Wayman S. DiTommaso A. Ryan M. (2021). Strip-tillage renovation of intermediate wheatgrass (*Thinopyrum intermedium*) for maintaining grain yield in mature stands. Renew. Agric. Food Syst. 36, 321–327. 10.1017/S1742170520000368

[B112] Law E. Wayman S. Pelzer C. J. Culman S. W. Gómez M. I. DiTommaso A. . (2022). Multi-criteria assessment of the economic and environmental sustainability characteristics of intermediate wheatgrass grown as a dual-purpose grain and forage crop. Sustainability 14, 3548. 10.3390/su14063548

[B113] Ledo A. Smith P. Zerihun A. Whitaker J. Vicente-Vicente J. L. Qin Z. . (2020). Changes in soil organic carbon under perennial crops. J. Glob. Chang. Biol. 26, 4158–4168. 10.1111/gcb.15120 32412147

[B114] Lemmon Z. H. Reem N. T. Dalrymple J. Soyk S. Swartwood K. E. Rodriguez-Leal D. . (2018). Rapid improvement of domestication traits in an orphan crop by genome editing. Nat. Plants 4, 766–770. 10.1038/s41477-018-0259-x 30287957

[B115] Li J. Zhou J. Zhang Y. Yang Y. Pu Q. Tao D. (2020). New insights into the nature of interspecific hybrid sterility in rice. Front. Plant Sci. 11, 555572. 10.3389/fpls.2020.555572 33072142 PMC7538986

[B116] Li S. Barreiro A. Jensen E. S. Zhang Y. Mårtensson L. M. D. (2020). Early interspecific dynamics, dry matter production and nitrogen use in Kernza (*Thinopyrum intermedium*)—alfalfa (*Medicago sativa* L.) mixed intercropping. Acta Agric. Scand. B Soil Plant Sci. 70, 165–175. 10.1080/09064710.2019.1686164

[B117] Li S. Gao F. Xie K. Zeng X. Cao Y. Zeng J. . (2016). The OsmiR396c-OsGRF4-OsGIF1 regulatory module determines grain size and yield in rice. Plant Biotechnol. J. 14, 2134–2146. 10.1111/pbi.12569 27107174 PMC5095787

[B118] Li T. Yang X. Yu Y. Si X. Zhai X. Zhang H. . (2018). Domestication of wild tomato is accelerated by genome editing. Nat. Biotechnol. 36, 1160–1163. 10.1038/nbt.4273 30272676

[B119] Li Z. Lathe R. S. Li J. He H. Bhalerao R. P. (2022). Toward understanding the biological foundations of perenniality. Trends Plant Sci. 27, 56–68. 10.1016/j.tplants.2021.08.007 34561180

[B120] Lindberg C. L. Hanslin H. M. Schubert M. Marcussen T. Trevaskis B. Preston J. C. . (2020). Increased above-ground resource allocation is a likely precursor for independent evolutionary origins of annuality in the Pooideae grass subfamily. New Phytol. 228, 318–329. 10.1111/nph.16666 32421861

[B121] Liston A. Wheeler J. A. (1994). The phylogenetic position of the genus Astragalus (Fabaceae): evidence from the chloroplast gene *rpoC1* and *rpoC2*. Biochem. Syst. Ecol. 22, 377–388. 10.1016/0305-1978(94)90028-0

[B122] Liu A. Burke J. M. (2006). Patterns of nucleotide diversity in wild and cultivated sunflower. Genetics 173, 321–330. 10.1534/genetics.105.051110 16322511 PMC1461453

[B123] Lowe K. La Rota M. Hoerster G. Hastings C. Wang N. Chamberlin M. . (2018). Rapid genotype “independent” *Zea mays* L. *(maize*) transformation *via* direct somatic embryogenesis. In Vitro Cell. Dev. Biol. Plant. 54, 240–252. 10.1007/s11627-018-9905-2 29780216 PMC5954046

[B124] Lowe K. Wu E. Wang N. Hoerster G. Hastings C. Cho M. J. . (2016). Morphogenic regulators Baby boom and Wuschel improve monocot transformation. Plant Cell 28, 1998–2015. 10.1105/tpc.16.00124 27600536 PMC5059793

[B125] Lundgren M. R. Des Marais D. L. (2020). Life history variation as a model for understanding trade-offs in plant-environment interactions. Curr. Biol. 10, R180–189. 10.1016/j.cub.2020.01.003 32097648

[B126] Lundqvist A. (1957). Self-incompatibility in rye: II. genetic control in the tetraploid. Hereditas 43, 467–511. 10.1111/j.1601-5223.1957.tb034~52.x

[B127] Luo G. Palmgren M. (2021). GRF-GIF chimeras boost plant regeneration. Trends Plant Sci. 26, 201–204. 10.10116/j.tplants.2020.12.001 33349565

[B128] Ma A. Qiu Y. Raihan T. Paudel B. Dahal S. Zhuang Y. . (2019). Genetics and genome-wide screening of regrowth loci, a key component of perennialism *in Zea diploperennis*. G3: Genes Genom. Genet. 9, 1393–1403. 10.1534/g3.118.200977 30808689 PMC6505134

[B129] Maher M. F. Nasti R. A. Vollbrecht M. Starker C. G. Clark M. D. Voytas D. F. (2020). Plant gene editing through de novo induction of meristems. Nat. Biotechnol. 38, 84–89. 10.1038/s41587-019-0337-2 31844292 PMC6954279

[B130] Manzanares C. Barth S. Thorogood D. Byrne S. L. Yates S. Czaban A. . (2016). A gene encoding a DUF247 domain protein cosegregates with the s self-incompatibility locus in perennial ryegrass. Mol. Biol. Evol. 33, 870–884. 10.1093/molbe v/msv335 26659250

[B131] McClure K. A. Sawler J. Gardner K. M. Money D. Myles S. (2014). Genomics: a potential panacea for the perennial problem. Am. J. Bot. 101, 1780–1790. 10.3732/ajb.1400143 25326620

[B132] McCown B. H. (2000). Special symposium: in vitro plant recalcitrance recalcitrance of woody and herbaceous perennial plants: dealing with genetic predeterminism. In Vitro Cell. Dev. Biol. Plant 36, 149–154. 10.1007/s11627-000-0030-6

[B133] Meijer R. (2020). Reimagining agriculture with perennial grains: a study on the diffusion of innovation and soil ecosystem services in the new perennial grain Kernza. Master's Thesis. Swedish University of Agricultural Sciences.

[B134] Mortenson J. S. Waldron B. L. Larson S. R. Jensen K. B. DeHaan L. R. Peel M. D. . (2019). Quantitative Trait Loci (QTL) for forage traits in intermediate wheatgrass when grown as spaced-plants vs. monoculture and polyculture swards. Agronomy 9, 580. 10.3390/agronomy9100580

[B135] Nemeth C. Yang C. Kasprzak P. Hubbart S. Scholefield D. Mehra S. . (2015). Generation of amphidiploids from hybrids of wheat and related species from the genera *Aegilops, Secale, Thinopyrum*, and *Triticum* as a source of genetic variation for wheat improvement. Genome 58, 71–79. 10.1139/gen-2015-0002 26053312

[B136] Norton M. R. Malinowski D. P. Volaire F. (2016). Plant drought survival under climate change and strategies to improve perennial grasses. a review. Agron. Sustain. Dev. 36, 29. 10.1007/s13593-016-0362-1

[B137] Olsen K. M. Wendel J. F. (2013). A bountiful harvest: genomic insights into crop domestication phenotypes. Annu. Rev. Plant Biol. 64, 47–70. 10.1146/annurev-arplant-050312-120048 23451788

[B138] Østerberg J. T. Xiang W. Olsen L. I. Edenbrandt A. K. Vedel S. E. Christiansen A. . (2017). Accelerating the domestication of new crops: feasibility and approaches. Trends Plant Sci. 22, 373–384. 10.1016/j.tplants.2017.01.004 28262427

[B139] Palmer N. A. Donze-Reiner T. Horvath D. Heng-Moss T. Waters B. Tobias C. . (2015). Switchgrass (*Panicum virgatum* L.) flag leaf transcriptomes reveal molecular signatures of leaf development, senescence and mineral dynamics. Funct. Integr. Genomics 15, 1–16. 10.1007/s10142-014-0393-0 25173486

[B140] Parry M. A. J. Madgwick P. J. Bayon C. Tearall K. Hernandez-Lopez A. Baudo M. . (2009). Mutation discovery for crop improvement. J. Exp. Bot. 60, 2817–2825. 10.1093/jxb/erp189 19516074

[B141] Paterson A. H. Kong W. Johnston R. M. Nabukalu P. Wu G. Poehlman W. L. . (2020). The evolution of an invasive plant, *Sorghum halepense* L. ('Johnsongrass'). Front. Genet. 11, 317. 10.3389/fgene.2020.00317 32477397 PMC7240026

[B142] Paustian K. Lehmann J. Ogle S. Reay D. Robertson G. P. Smith P. (2016). Climate-smart soils. Nature 532, 49–57. 10.1038/nature17174 27078564

[B143] Peixoto L. Olesen J. E. Elsgaard L. Enggrob K. L. Banfield C. C. Dippold M. A. . (2022). Deep-rooted perennial crops differ in capacity to stabilize C inputs in deep soil layers. Sci. Rep. 12, 5952. 10.1038/s41598-022-09737-1 35396458 PMC8993804

[B144] Picasso V. D. Brummer E. C. Liebman M. Dixon P. M. Wilsey B. J. (2008). Crop species diversity affects productivity and weed suppression in perennial polycultures under two management strategies. Crop Sci. 48, 331–342. 10.2135/cropsci2007.04.0225

[B145] Pickering R. (1991). The production of fertile triploid hybrids from crosses between *Hordeum vulgare* L. (2n = 4*x* = 28) and *H. bulbosum* L. (2n = 2*x* = 14). Hereditas 114, 227–236. 10.1111/j.1601-5223.1991.tb00329.x

[B146] Pinto R. S. Lopes M. S. Collins N. C. Reynolds M. P. (2016). Modeling and genetic dissection of staygreen under heat stress. Theor. Appl. Genet. 129, 2055–2074. 10.1007/s00122-016-2757-4 27545985 PMC5069319

[B147] Rahaman M. M. Chen D. Gillani Z. Klukas C. Chen M. (2015). Advanced phenotyping and phenotype data analysis for the study of plant growth and development. Front. Plant Sci. 6, 619. 10.3389/fpls.2015.00619 26322060 PMC4530591

[B148] Rakszegi M. Kisgyörgy B. N. Tearell K. Shewry P. Làng L. Phillips A. . (2010). Diversity of agronomic and morphological traits in a mutant population of bread wheat studied in the Healthgrain program. Euphytica 174, 409–421. 10.1007/s10681-010-0149-4

[B149] Ramírez-González R. H. Borrill P. Lang D. Harrington S. A. Brinton J. Venturini L. . (2018). The transcriptional landscape of polyploid wheat. Science 361, eaar6089. 10.1126/science.aar6089 30115782

[B150] Reiss E. R. Drinkwater L. E. (2018). Cultivar mixtures: a meta-analysis of the effect of intraspecific diversity on crop yield. Ecol. Appl. 28, 62–77. 10.1002/eap.1629 28940830

[B151] Reynolds M. P. Acevedo E. Sayre K. D. Fischer R. A. (1994). Yield potential in modern wheat varieties: its association with a less competitive ideotype. Field Crops Res. 37, 149–160. 10.1016/0378-4290(94)90094-9

[B152] Ryan M. R. Crews T. E. Culman S. W. DeHaan L. R. Hayes R. C. Jungers J. M. . (2018). Managing for multifunctionality in perennial grain crops. Bioscience 68, 294–304. 10.1093/biosci/biy014 29662249 PMC5894082

[B153] Sacks E. J. McNally K. M. Liu L. Lafitte R. Cruz T. S. (2008). Genetic variation for perenniality in *O. sativa/O.rufipogon derivatives. Adv. Rice Genet*. 123–125. 10.1142/9789812914319_0049

[B154] Sacks E. J. Roxas J. P. Cruz M. T. S. (2003). Developing perennial upland rice II: Field performance of S1 families from an intermated *Oryza sativa*/O. longistaminata population. Crop Sci. 43, 129–134. 10.2135/cropsci2003.1290

[B155] Schaart J. G. van de Wiel C. C. M. Lotz L. A. P. Smulders M. J. M. (2016). Opportunities for products of new plant breeding techniques. Trends Plant Sci. 21, 438–449. 10.1016/j.tplants.2015.11.006 26654659

[B156] Schlautman B. Barriball S. Ciotir C. Herron S. Miller A. J. (2018). Perennial grain legume domestication Phase 1: criteria for candidate species selection. Sustainability. 10, 730. 10.3390/su10030730

[B157] Shinozuka H. Cogan N. O. I. Smith K. F. Spangenberg G. C. Forster J. W. (2010). Fine-scale comparative genetic and physical mapping supports map-based cloning strategies for the self-incompatibility loci of perennial ryegrass (*Lolium perenne* L.). Plant Mol. Biol. 72, 343–355. 10.1007/s1110~3-009-9574-y 19943086

[B158] Soto-Gómez D. Pérez-Rodríguez P. (2022). Sustainable agriculture through perennial grains: wheat, rice, maize, and other species. A review. Agricult. Ecosyst. Environ. 325, 107747. 10.1016/j.agee.2021.107747

[B159] Spano G. Di Fonzo N. Perrotta C. Platani C. Ronga G. Lawlor D. W. . (2003). Physiological characterization of ‘stay green' mutants in durum wheat. J. Exp. Bot. 54, 1415–1420. 10.1093/jxb/erg150 12709488

[B160] Sprunger C. D. Martin T. Mann M. (2020). Systems with greater perenniality and crop diversity enhance soil biological health. Agric. Environ. Lett. 10.1002/ael2.20030

[B161] Swentowsky K. W. Bell H. S. Wills D. M. Kelly Dawe R. (2021). QTL map of early- and late-stage perennial regrowth in *Zea diploperennis*. Front. Plant Sci. 12, 707839. 10.3389/fpls.2021.707839 34504508 PMC8421791

[B162] Tao D. Y. Sripichitt P. (2000). Preliminary report on transfer traits of vegetative propagation from wild rice species to *Oryza sativa via* distant hybridization and embryo rescue. Kasetsart J. (Nat. Sci.) 34, 1–11. Available online at: https://li01.tci-thaijo.org/index.php/anres/article/view/240369/163918

[B163] Tautges N. E. Jungers J. M. Dehaan L. R. Wyse D. L. Sheaffer C. C. (2018). Maintaining grain yields of the perennial cereal intermediate wheatgrass in monoculture v. bi-culture with alfalfa in the Upper Midwestern USA. J. Agricult. Sci. 156, 758–773. 10.1017/S0021859618000680

[B164] Thomas H. Ougham H. (2014). The stay-green trait. J. Exp. Bot. 65, 3889–3900. 10.1093/jxb/eru037 24600017

[B165] Thomas H. Thomas H. M. Ougham H. (2000). Annuality, perenniality and cell death. J. Exp. Bot. 51, 1781–1788. 10.1093/jexbot/51.352.1781 11113157

[B166] Thorsted M. D. Weiner J. Olesen J. E. (2006). Above- and below- ground competition between intercropped winter wheat *Triticum aestivum* and white clover *Trifolium repens*. J. Appl. Ecol. 43, 237–245. 10.1111/j.1365-2664.2006.01131.x

[B167] Tilman D. (1988). Plant strategies and the dynamics and structure of plant communities. Monographs in Population Biology 26, 126-129. Princeton University Press. 10.1515/9780691209593

[B168] Tork D. G. Anderson N. O. Wyse D. L. Betts K. J. (2019). Domestication of perennial flax using an ideotype approach for oilseed, cut flower, and garden performance. Agronomy 9, 707. 10.3390/agronomy9110707

[B169] USDA (2021). Crop Explorer—World Agricultural Production (WAP) Briefs—Europe. Foreign Agricultural Service, U. S. Department of Agriculture. Available online at: https://ipad.fas.usda.gov/cropexplorer/pecad_stories.aspx?regionid=europeandftype=prodbriefs (accessed May 17, 2022).

[B170] Van Tassel D. L. Albrecht K. A. Bever J. D. Boe A. A. Brandvain Y. Crews T. E. . (2017). Accelerating *Silphium* domestication: an opportunity to develop new crop ideotypes and breeding strategies informed by multiple disciplines. Crop Sci. 57, 1274–1284. 10.2135/cropsci2016.10.0834

[B171] Verboom G. A. Linder H. P. Stock W. D. (2004). Testing the adaptive nature of radiation: growth form and life history divergence in the African grass genus *Ehrharta* (Poaceae: Ehrhartoideae). Am. J. Bot. 91, 1364–1370. 10.3732/ajb.91.9.1364 21652369

[B172] von Bothmer R. Jacobsen N. Baden C. Jorgensen R. B. Linde-Laursen I. (1995). An ecogeographical study of the genus Hordeum. 2nd edition. Systematic and Ecogeographic Studies on Crop Genepools 7. Rome: international Plant Genetic Resources Institute.

[B173] Wang B. Regulski M. Tseng E. Olson A. Goodwin S. McCombie W. R. . (2018). A comparative transcriptional landscape of maize and sorghum obtained by single-molecule sequencing. Genome Res. 28, 921–932. 10.1101/gr.227462.117 29712755 PMC5991521

[B174] Wang K. Shi L. Liang X. Zhao P. Wang W. Liu J. . (2022). The gene TaWOX5 overcomes genotype dependency in wheat genetic transformation. Nat. Plants 10.1038/s41477-021-01085-8 35027699

[B175] Wang R. Farrona S. Vincent C. Joecker A. Schoof H. Turck F. . (2009). PEP1 regulates perennial flowering in *Arabis alpina*. Nature 21, 423–427. 10.1038/nature07988 19369938

[B176] Washburn J. D. Murray S. C. Burson B. L. Klein R. R Jessup R. W. (2013). Targeted mapping of quantitative trait locus regions for rhizomatousness in chromosome SBI-01 and analysis of overwintering in a *Sorghum bicolor* x *S. propinquum population*. Mol. Breed. 31, 153–162. 10.1007/s11032-012-9778-8 23316113 PMC3538016

[B177] Watson A. Ghosh S. Williams M. J. Cuddy W. Simmonds J. R. Rey M. D. . (2018). Speed breeding is a powerful tool to accelerate crop research and breeding. Nat. Plants 4, 23–29. 10.1038/s41477-017-0083-8 29292376

[B178] Weißhuhn P. Reckling M. Stachow U. Wiggering H. (2017). Supporting agricultural ecosystem services through the integration of perennial polycultures into crop rotations. Sustainability 9, 2267. 10.3390/su9122267

[B179] Wendler N. Mascher M. Himmelbach A. Bini F. Kumlehn J. Stein N. (2017). A high-density, sequence-enriched genetic map of *Hordeum bulbosum* and its collinearity to H. vulgare. Plant Genome 10, 3. 10.3835/plantgenome2017.06.0049 29293821

[B180] Wendler N. Mascher M. Himmelbach A. Johnston P. Pickering R. Stein N. (2015). Bulbosum to go: a toolbox to utilize *Hordeum vulgare*/*bulbosum* introgressions for breeding and beyond. Mol. Plant 8, 1507–1519. 10.1016/j.molp.2015.05.004 25983208

[B181] Westerbergh A. Doebley J. (2004). Quantitative trait loci controlling phenotypes related to the perennial vs. annual habit in wild relatives of maize. Theor. Appl. Genet. 109, 1544–1553. 10.1007/s00122-004-1778-6 15338134

[B182] Westerbergh A. Lerceteau-Köhler E. Sameri M. Bedada G. Lundquist P.-O. (2018). Toward the development of perennial barley for cold temperate climates—Evaluation of wild barley relatives as genetic resources. Sustainability 10, 1969. 10.3390/su10061969

[B183] Yahya M. S. Syafiq M. Ashton-Butt A. Ghazali A. Asmah S. Azhar B. (2017). Switching from monoculture to polyculture farming benefits birds in oil palm production landscapes: evidence from mist netting data. Ecol. Evol. 7, 6314–6325. 10.1002/ece3.3205 28861235 PMC5574735

[B184] Yang W. Feng H. Zhang X. Zhang J. Doonan J. H. Batchelor W. D. . (2020). Crop phenomics and high-throughput phenotyping: past decades, current challenges, and future perspectives. Mol. Plant 13, 187–214. 10.1016/j.molp.2020.01.008 31981735

[B185] Yates S. Jaškun,e K. Liebisch F. Nagelmüller S. Kirchgessner N. Kölliker R. . (2019). Phenotyping a dynamic trait: leaf growth of perennial ryegrass under water limiting conditions. Front. Plant Sci. 10, 344. 10.3389/fpls.2019.00344 30967891 PMC6440318

[B186] Yu H. Lin T. Meng X. Du H. Zhang J. Liu G. . (2021). A route to de novo domestication of wild allotetraploid rice. Cell 184, 1156–1170. 10.1016/j.cell.2021.01.013 33539781

[B187] Yuan R. Zhao N. Usman B. Luo L. Liao S. Qin Y. . (2020). Development of chromosome segment substitution lines (CSSLs) derived from Guangxi wild rice (*Oryza rufipogon* Griff.) under rice (*Oryza sativa* L.) background and the identification of QTLs for plant Architecture, agronomic traits and cold tolerance. Genes 11, 980. 10.3390/genes11090980 32842674 PMC7564255

[B188] Zhang S. Huang G. Zhang J. Huang L. Cheng M. Wang Z. . (2019). Genotype by environment interactions for performance of perennial rice genotypes (*Oryza sativa* L./*Oryza longistaminata*) relative to annual rice genotypes over regrowth cycles and locations in southern China. Field Crops Res. 241, 107556. 10.1016/j.fcr.2019.107556

[B189] Zhang X. Larson S. R. Gao L. The S. L. DeHaan L. R. Fraser M. (2017). Uncovering the genetic architecture of seed weight and size in intermediate wheatgrass through linkage analysis and association mapping. Plant Genome 10, 1–15 10.3835/plantgenome2017.03.0022 29293813

[B190] Zhang X. Sallam A. Gao L. Kantarski T. Poland J. A. Wyse D. L. . (2016). Establishment and optimization of genomic selection to accelerate the domestication and improvement of intermediate wheatgrass. Plant Genome 9, 1–18. 10.3835/plantgenome2015.07.0059 27898759

[B191] Zillinsky F. J. (1973). Triticale breeding and research at CIMMYT: a progress report. Res. Bull. 24, 1–84.

[B192] Zsögön A. Cermák T. Naves E. R. Notini M. M. Edel K. H. Weinl S. . (2018). De novo domestication of wild tomato using genome editing. Nat. Biotechnol. 36, 1211–1216. 10.1038/nbt.4272 30272678

